# Synthesis, anti-leishmanial and molecular docking study of bis-indole derivatives

**DOI:** 10.1186/s13065-019-0617-4

**Published:** 2019-08-06

**Authors:** Muhammad Taha, Imad Uddin, Mohammed Gollapalli, Noor Barak Almandil, Fazal Rahim, Rai Khalid Farooq, Muhammad Nawaz, Mohamed Ibrahim, Mohammed A. Alqahtani, Yasser A. Bamarouf, Manikandan Selvaraj

**Affiliations:** 1 Department of Clinical Pharmacy, Institute for Research and Medical Consultations (IRMC), Imam Abdulrahman Bin Faisal University, P.O. Box 1982, Dammam, 31441 Saudi Arabia; 2 Department of Chemistry, Hazara University, Mansehra, 21300 Khyber Pakhtunkhwa Pakistan; 3 Department of Computer Information Systems, College of Computer Science & Information Technology, Imam Abdulrahman Bin Faisal University, P. O. Box 1982, Dammam, 31441 Saudi Arabia; 4 Department of Neuroscience Research, Institute of Research and Medical Consultations, Imam Abdulrahman Bin Faisal University, P.O. Box 1982, Dammam, 31441 Saudi Arabia; 5 Department of Nano-Medicine Research, Institute for Research and Medical Consultations (IRMC), Imam Abdulrahman Bin Faisal University, P.O. Box 1982, Dammam, 31441 Saudi Arabia; 6 Monash University School of Chemical Engineering, 47500 Bandar Sunway, Selangor Malaysia

**Keywords:** Synthesis, Bisindole, Leishmaniasis, Molecular docking, SAR

## Abstract

We have synthesized new series of bisindole analogs (**1**–**27**), characterized by ^1^HNMR and HR-EI-MS and evaluated for their anti-leishmanial potential. All compounds showed outstanding inhibitory potential with IC_50_ values ranging from 0.7 ± 0.01 to 13.30 ± 0.50 µM respectively when compared with standard pentamidine with IC_50_ value of 7.20 ± 0.20 µM. All analogs showed greater potential than standard except **10**, **19** and **23** when compared with standard. Structure activity relationship has been also established for all compounds. Molecular docking studies were carried out to understand the binding interaction of active molecules.

**Electronic supplementary material:**

The online version of this article (10.1186/s13065-019-0617-4) contains supplementary material, which is available to authorized users.

## Introduction

Leishmaniasis has affected almost 98 countries of the world. Every year approximately in 2 million people leishmaniasis has been reported while 350 million people are at risk [1]. The efficacy of drugs available for leishmaniasis is limited [2]. Leishmaniasis, a parasitic disease unveiled by four syndromes which are cutaneous leishmaniasis, visceral leishmaniasis, muco cutaneous leishmaniasis and kalaazar dermal leishmaniasis. In 90% population of India, Bangladesh, Nepal, East Africa and Brazil visceral leishmaniasis have been reported. The first-line drugs used for the treatment of leishmaniasis are pentavalent antimonial compounds which is not too much effective in almost 60% cases due to drug resistance. Some other treatment has been introduced for visceral leishmaniasis which has serious limitation [3]. Some second line drugs are also used for the treatment like pentamidine amphotericin B, but they have toxicity problems and unavailability [4, 5]. Some vaccine has been introduced for leishmaniasis infections which are effective with low price however effective vaccine is not yet introduce [6, 7]. The growth and survival of leishmanial parasite depend on polyamine bases which are mainly produce during metabolic process. Interaction directly with polyamine or biosynthetic pathways of these bases could result in leishmanial infection [8]. The most challenging task is the introduction of an affordable, effective and alternative antilieshmanial drug.

Bisindole compounds are known to have wide range of pharmacological activities like anticancer and antimicrobial [9–14], etc. Hamacanthin A bisindole alkaloid isolated from the sponge *Hamacantha* sp. and *Spongosorites* sp. exhibited effective antibacterial activity against *Staphylococcus aureus* and MRS with MIC of 6.45 mM and antifungal activity against *Bacillus subtilis* with MIC of 3.22 mM [15–18]. Additionally, bisindoles compounds have been used in many biological processes such as fluorescent molecular probes [19]. Amongst several antileishmanial scaffolds reported, indole alkaloids [20–25] showed promising activity against Leishmania parasite.

Keeping the idea for designing of new antilieshmanial drug, it is important to synthesize molecules having different biological properties based on their structure domain. We have synthesized variety of biologically active compounds for specific biological target [26–31]. Herein we report the synthesis of bis-indole derivatives as antilieshmanial agents.

## Results and discussions

### Chemistry

**Scheme 1:**
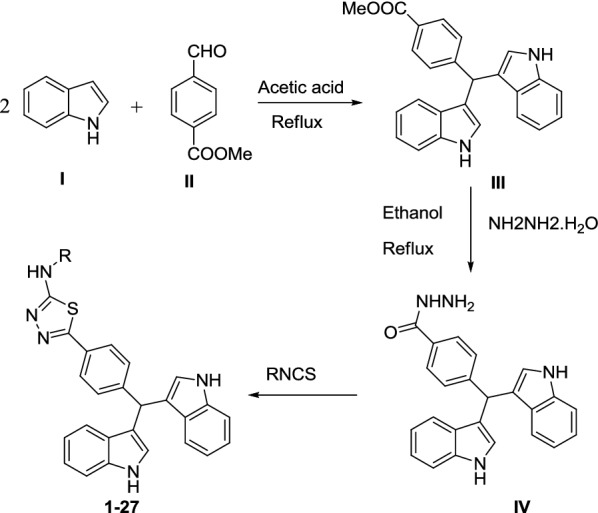
Synthesis of *bis*-indole derivatives (**1**–**27**)

The synthesis of bis-indole analogs (**1**–**27**) was carried out in three steps. In the first step, 2 equivalent of indole (**I**) was mixed with methyl-4-formylbenzoate (**II**) in acetic acid and reflux for 4–6 h to afford intermediate product **III**. The Intermediate **III** was then treated with hydrazine hydrate (3 mL) in ethanol, then reflux for 3–4 h to obtained intermediate **IV**. The intermediate **IV** was then mixed with different isothiocyanates to get the pure products (**1**–**27**) in good yield. All reactions completion was monitored by periodic TLC. Structures of all synthesized analogs were confirmed with ^1^HNMR, ^13^CNMR and HR-EIMS (Scheme 1).

### Biological activity

In the continuation of our effort for enzyme inhibition [32–36], we have synthesized series of bisindole derivatives as a new class of anti-leishmanial agents. All compounds (**1**–**27**) were screened for their leishmanial activity (Table 1). All these compounds showed outstanding inhibition when compared with standard. Out of **27** analogs, fifteen compounds i.e. **3**, **4**, **7**, **8**, **9**, **11**, **12**, **15**, **16**, **17**, **18**, **20**, **21**, **22** and **25** showed excellent inhibitory potential with IC_50_ values ranging from 0.7 ± 0.01 to 4.30 ± 0.20 µM respectively when compared with standard pentamidine having IC_50_ value of 7.20 ± 0.20 µM. Compounds **1**, **2**, **5**, **6**, **10**, **13**, **14**, **19**, **23**, **24**, **26** and **27** also showed excellent inhibition ranging from 5.20 ± 0.2 to 13.30 ± 0.50 µM when compared with standard.

**Table 1:**
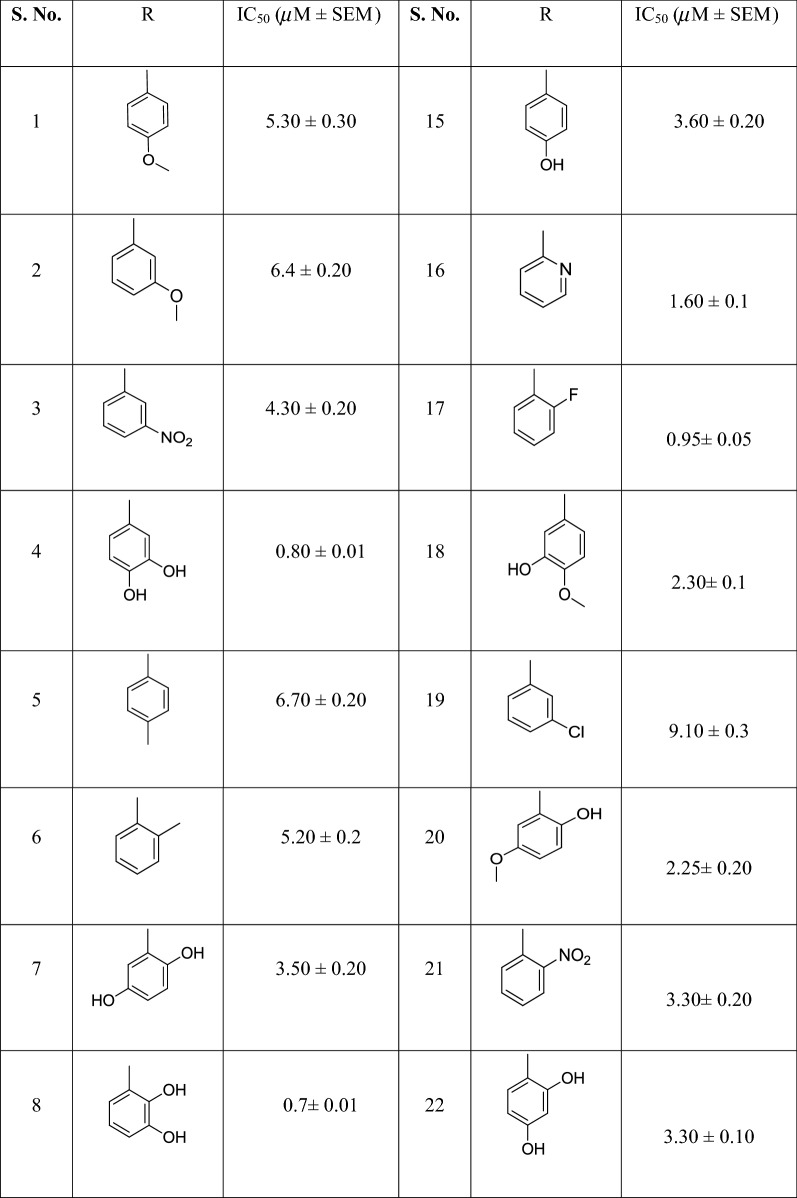

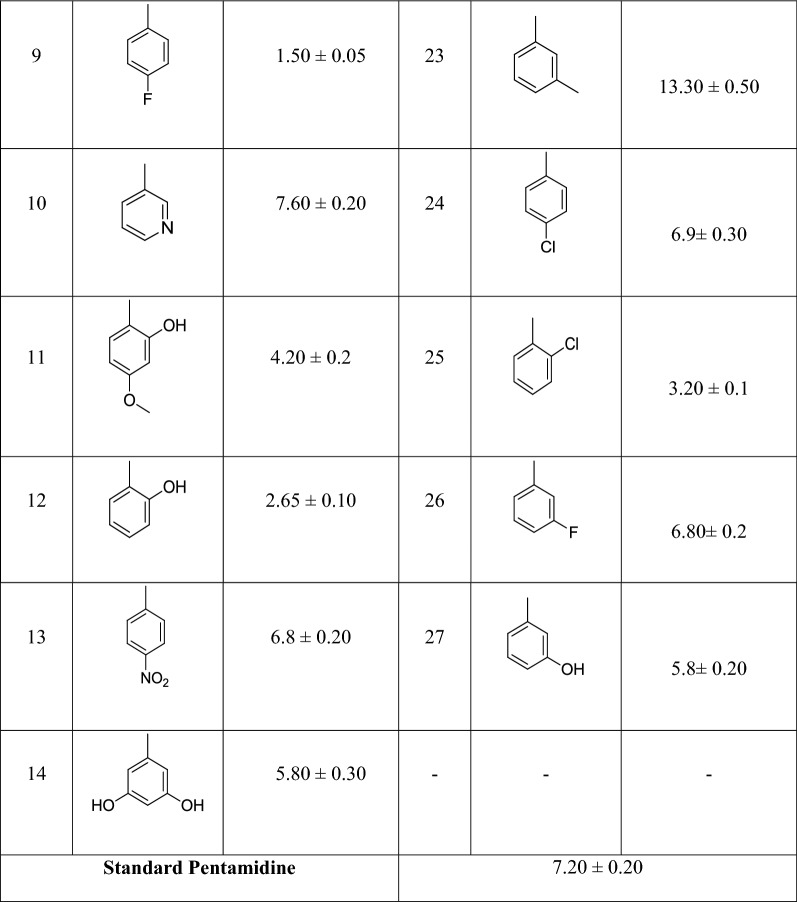
Different constituents of bis-indole and their anti-leishmanial potential

*SEM* standard error mean

Structure activity relationship (SAR) has been established for all compounds. The compound **8**, a 2,3-dihydroxy analog was found to be the most potent among the series with (IC_50_ value 0.7 ± 0.01 µM). If we compare analog **8** with other dihydroxy analogs **4**, a 2,3-dihydroxy (IC_50_ value 0.80 ± 0.01 µM) **7**, a 2,5-dihydroxy (IC_50_ values 3.50 ± 0.20) **14**, a 3,5-dihydroxy analog (IC_50_ values 5.80 ± 0.30), and **22** a 2,4-dihydroxy analogs (IC_50_ values 3.30 ± 0.10) it’s clear that vicinal dihydroxy system i.e. **8** and **4** showed excellent inhibitory potential rather as compared the other dihydroxy analogs. This indicates the vicinal dihydroxy system is conjugated effectively with enzyme Pteridine reductase to cause higher inhibition. Comparing analogs **12**, **15** and **27**, monohydroxy analogs the 2-hydroxy analog **12** (IC_50_ value 2.65 ± 0.10 µM) is more potent than 3-hydroxy and 4-hydroxy analogs **15** (IC_50_ value 3.60 ± 0.20 µM) and **27** (IC_50_ value 5.8 ± 0.20 µM) showing its effective binding with enzyme. Compound **6** with 2-methyl on phenyl ring showed good active (IC_50_ value 5.2 0 ± 0.2 µM). The methyl may be involved in interaction through inductive effect. The compound **1** having 4-methoxy showed better activity than compound **2** having 3-methoxy with IC_50_ value 5.30 ± 0.30 and 6.4 ± 0.20 µM respectively. The 2-nitro analog **21** (IC_50_ value 3.30 ± 0.20 µM) is more potent when compared with 3-nitro analog **3** (IC_50_ value 4.30 ± 0.20 µM) and 4-nitro analog **13** (IC_50_ value 6.8 ± 0.20 µM). This shows that position of substituents plays a vital role in inhibition. *Ortho* fluoro analog **17** (IC_50_ value 0.95 ± 0.05 µM) is much superior than *meta* and *para* fluoro analogs **9** and **26** with IC_50_ values 1.50 ± 0.05, and 6.80 ± 0.2 µM respectively. So, it was concluded from this study that the nature, position and number of substituents play a critical role in the inhibitory potential of our designed analogs Table 1.

### Molecular docking studies of bis-indole derivatives on pteridine reductase

**Fig. 1:**
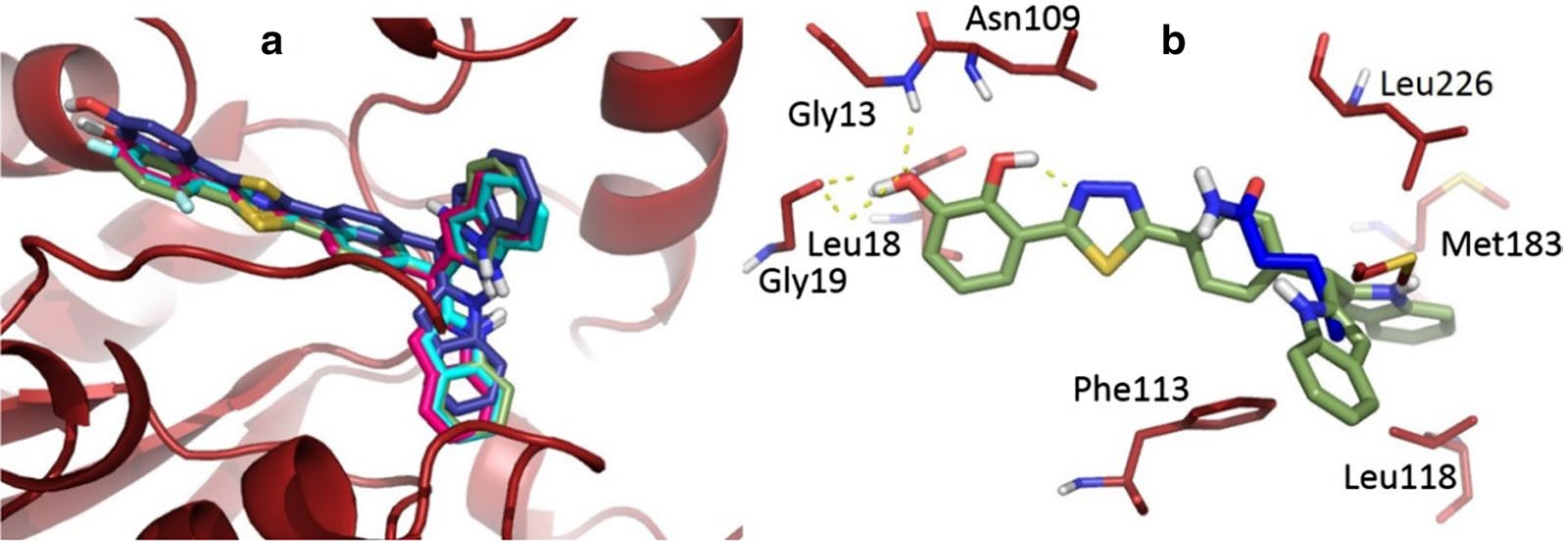
**a** Shows the binding mode of the four most active compounds in pteridine reductase active site. **b** Binding mode of compound **8** (green color) in comparison with pentamidine (blue color)

Docking studies with PTR shows that all the active compounds tend to adopt a similar binding mode as depicted in Fig. 1a. Comparison of the binding mode of the most active compound **8** with standard pentamidine used in the study, shows that the compound **8** interacts with the key residues of the PTR active site establishing hydrophilic and hydrophobic contacts, while in the case of the pentamidine interacts with fewer hydrophobic residues as shown in Fig. 1b. This clearly shows that this class of synthetic derivatives could be potential candidates for therapeutic against leishmaniasis.

**Fig. 2:**
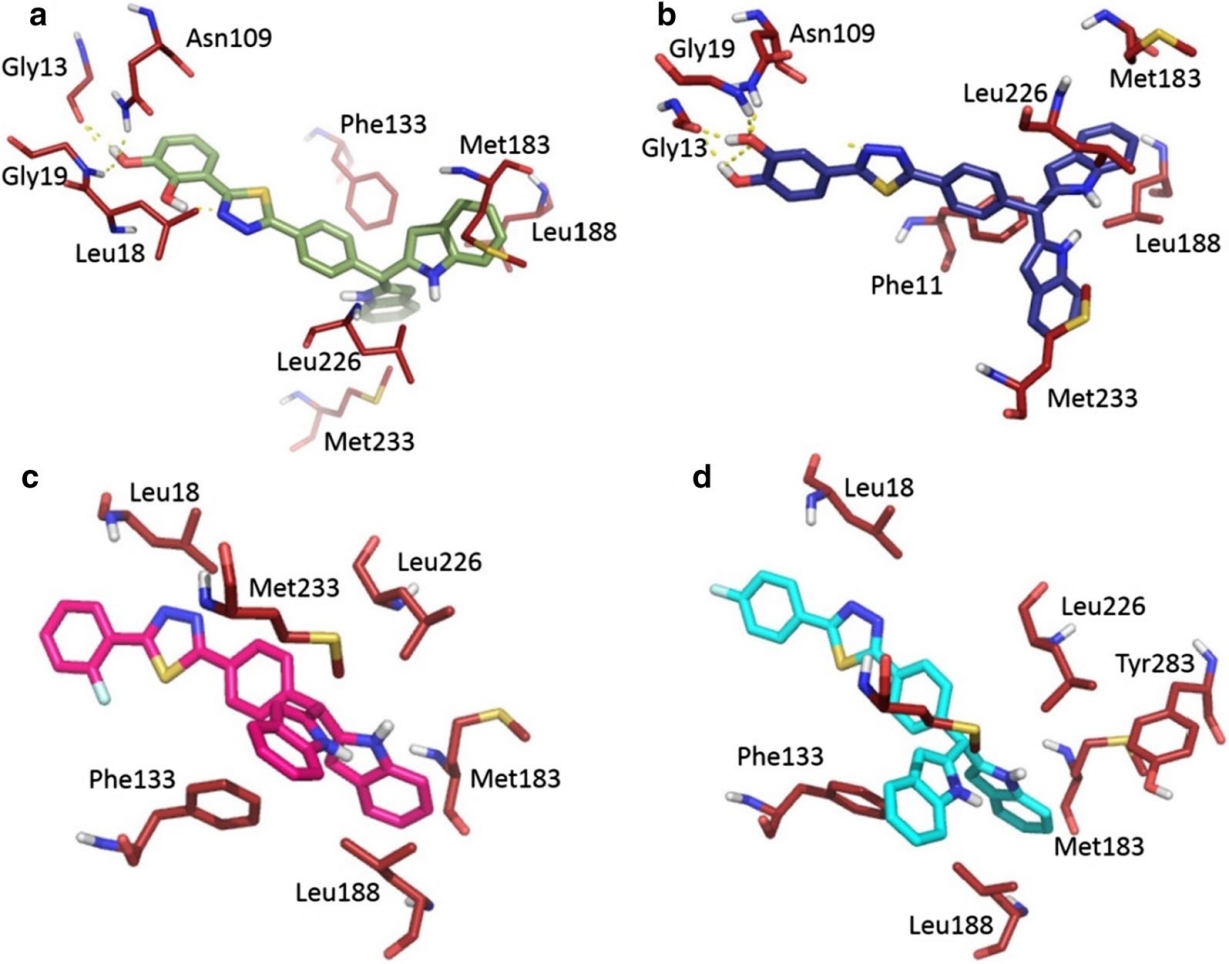
Shows the binding mode of **a** compound **8**, **b** compound **4**, **c** compound **17**, and **d** compound **9** in pteridine reductase active site. Hydrogen bonds are represented in dashed yellow lines and the key interacting restudies are represented in line form

The activity profile of these derivatives ranges from IC_50_ (0.7 μM to 13.30 μM). Therefore, it’s clear that these compounds are good starting point in pteridine reductase inhibitor discovery. In the following section, we limit our self to report only the binding mode of four most active compounds. Binding mode of compound **8** (Fig. 2a) shows that the *meta* hydroxy group attached to the benzene ring forms hydrogen bonds with side chain of Gly13, Gly19 and Asn109, respectively. While the phenyl ring positioned at compound’s center forms π-π stacking with Phe133. In addition, the di-indole rings form hydrophobic interaction with residues such as Met183, Leu188, Met233 and Leu226, respectively.

Figure 2b shows the binding mode of compound **4**, where the *meta* and *para* positioned hydroxy moieties forms hydrogen bond with side chains of Gly13, Gly19 and Asn109. Next, the phenyl ring positioned at the center of the compound forms π–π stacking with Phe11 and the di-indole rings forms hydrophobic interaction with residues such as Met183, Leu188, Met233 and Leu226 similarly as in case of compound **8**. Interestingly, in the case of compound **17**, the entire complex was stabilized by hydrophobic interaction. The *2*-fluorobenzene group forms hydrophobic contact with Leu18 and the phenyl ring forms π–π stacking with Phe133 and hydrophobic contact with Met233, respectively. Finally, the di-indole rings form non-polar contact with Met183, Leu188 and Leu226. Likewise, the compound **9** forms hydrophobic contacts with 3-fluorobenzene group with Leu18 and the phenyl ring forms π–π stacking with Phe133 and the di-indole rings interaction with Met183, Leu188, Leu226 and Tyr283 stabilize the complex.

### Conclusion

It was concluded from this study that a series of bisindole analogs (**1**–**27**) were synthesized, characterized by ^1^HNMR and HR-EI-MS and evaluated for their anti-leishmanial potential. All compounds showed outstanding inhibitory potential with IC_50_ values ranging from 0.7 to 13.30 µM respectively when compared with standard pentamidine with IC_50_ value of 7.20 ± 0.20 µM. Structure activity relationship has been also established for all compounds, which shows that the nature, position and number of substituents on phenyl ring play a critical role. Molecular docking studies were carried out to understand the binding interaction of our synthesized molecules with the active site of this enzyme (Additional file 1).

## Materials and methods

NMR experiments were performed on Avance Bruker AM 300 MHz machine. Electron impact mass spectra (EI MS) were recorded on a Finnigan MAT-311A (Germany) mass spectrometer. Thin layer chromatography (TLC) was performed on pre-coated silica gel aluminum plates (Kieselgel 60, 254, E. Merck, Germany). Chromatograms were visualized by UV at 254 and 365 nm.

### Molecular docking studies

In this recent work, we have used pteridine reductase (PTR) as vital drug target against leishmaniasis, a vital enzyme accountable for pteridine salvage in leishmania protozoans. For the molecular docking studies, we have used similar protocol that has been adopted in our previous work for both ligand preparation and docking studies of derivatives of bis-indole against PTR. Molecular docking studies were carried out using glide: a complete solution for ligand-receptor docking in small molecule drug discovery suite. Initially, receptor grid generation was done by generating grid on the Pteridine reductase structure were the grid box was centered on methotrexate (MTX) complexed ligand with 12 Å radius respectively. Both standards precision (SP) mode and extra precision (XP) mode was chosen during the Glide docking process and Glide score was considered for analysis. Further top rank scored binding mode analyzed in Pymol [37].

### General procedure for the synthesis of compounds (**1**–**27**)

The synthetic scheme towards the synthesis of bis-indole compounds involved mixing of indole with methyl-4-formylbenzoate in acetic acid to afford the ester intermediate which was then reacted with hydrazine hydrate and finally with isothiocyanate to get the final products **1**–**27**.

The synthesis of bis-indole analogs (**1**–**27**) was carried out in three steps. In the first step, 2 equivalent of indole (**I**) was mixed with methyl-4-formylbenzoate (**II**) in acetic acid and reflux for 4–6 h. to afford intermediate product **III**. The Intermediate **III** was then treated with hydrazine hydrate (3 mL) in ethanol, then reflux for 3–4 h to obtained intermediate **IV**. The intermediate **IV** was then mixed with different isothiocyanates to get the pure products (**1**–**27**) in good yield. All reaction completion was monitored by periodic TLC. Structures of all synthesized analogs were confirmed with ^1^HNMR, ^13^CNMR and HR-EIMS.

#### 5-(4-(Di(1*H*-indol-3-yl)methyl)phenyl)-*N*-(4-methoxyphenyl)-1,3,4-thiadiazol-2-amine (**1**)

Yield 90%, ^1^H-NMR (500 MHz, DMSO-*d*_*6*_): *δ* 12.30 (s, 2H, NH), 11.60 (s, 1H, NH), 7.74 (d, *J* = 7.5 Hz, 2H, Ar), 7.52 (d, *J* = 7.4 Hz, 2H, Ar), 7.48 (d, *J* = 7.3 Hz, 2H, Ar), 7.20 (d, *J* = 7.0 Hz, 2H, Ar), 7.00 (d, *J* = 7.1 Hz, 2H, Ar), 6.83 (dd, *J* = 8.2, 2.5 Hz, 2H, Ar), 6.70 (dd, *J* = 7.9, 3.2 Hz, 2H, Ar), 6.61 (d, *J* = 7.0 Hz, 4H, Ar), 6.21 (H, CH), 3.83 (s, 3H, CH_3_); ^13^C-NMR (125 MHz, DMSO-*d*_*6*_): *δ* 174.3, 153.1, 152.2, 138.4, 136.1, 136.2, 133.0, 130.2, 129.2, 129.2, 127.2, 127.2, 127.2, 127.2, 123.3, 123.1, 121.9, 121.4, 121.3, 121.1, 119.5, 119.1, 118.6, 118.4, 115.3, 115.2, 112.6, 112.3, 111.4, 111.2, 55.4, 54.2; HR-EIMS: m/z calcd for C_32_H_25_N_5_OS [M]^+^ 527.1780, Found 527.1768.

#### 5-(4-(Di(1*H*-indol-3-yl)methyl)phenyl)-*N*-(3-methoxyphenyl)-1,3,4-thiadiazol-2-amine (**2**)

Yield 82%, ^1^H-NMR (500 MHz, DMSO-*d*_*6*_): *δ* 10.52 (s, 2H, NH), 10.12 (s, 1H, NH), 7.76 (d, *J* = 7.5 Hz, 2H, Ar), 7.52–7.49 (m, 3H, Ar), 7.47 (d, *J* = 7.3 Hz, 2H, Ar), 7.42–7.40 (m, 1H, Ar), 7.22 (dd, *J* = 8.0, 3.0 Hz, 1H, Ar), 7.20 (d, *J* = 7.5 Hz, 2H, Ar), 6.85 (dd, *J* = 8.0, 2.0 Hz, 2H, Ar), 6.80 (d, *J* = 7.5 Hz, 1H, Ar), 6.70 (dd, *J* = 7.5, 3.0 Hz, 2H, Ar), 6.58 (d, *J* = 7.0 Hz, 2H, Ar), 6.20 (s, 1H, CH), 3.90 (s, 3H, OCH_3_); ^13^C-NMR (125 MHz, DMSO-*d*_*6*_): *δ* 174.3, 152.5, 142.1, 139.0, 138.0, 136.3, 136.2, 130.2, 129.8, 129.4, 129.1, 127.7, 127.5, 127.3, 127.1, 123.4, 123.2, 121.7, 121.5, 121.3, 119.5, 119.3, 119.1, 118.6, 118.3, 114.4, 112.4, 112.2, 111.4, 111.2, 54.3, 61.2; HR-EIMS: m/z calcd for C_32_H_25_N_5_OS [M]^+^ 527.178, Found 527.168.

#### 5-(4-(Di(1*H*-indol-3-yl)methyl)phenyl)-*N*-(3-nitrophenyl)-1,3,4-thiadiazol-2-amine (**3**)

Yield 79%, ^1^H-NMR (500 MHz, DMSO-*d*_*6*_): *δ* 12.60 (s, 2H, NH), 12.10 (s, 1H, NH), 8.10 (s, 1H), 8.00 (d, *J* = 7.8 Hz, 1H, Ar), 7.75 (d, *J* = 7.5 Hz, 2H, Ar), 7.70–7.65 (m, 3H, Ar), 7.48 (d, *J* = 7.3 Hz, 2H, Ar), 7.45 (dd, *J* = 8.2 3.4 Hz, 1H, Ar), 7.20 (d, *J* = 7.0 Hz, 2H, Ar), 6.82 (dd, *J* = 8.2, 2.5 Hz, 2H, Ar), 6.68 (dd, *J* = 7.9, 3.2 Hz, 2H, Ar), 6.62 (d, *J* = 7.0 Hz, 2H, Ar), 6.21 (s, 1H, CH); ^13^C-NMR (125 MHz, DMSO-*d*_*6*_): *δ* 174.3, 152.4, 148.3, 143.1, 138.4, 136.3, 136.2, 130.3, 130.2, 129.2, 129.1, 127.1, 127.0, 127.0, 126, 123.7, 123.5, 123.2, 121.5, 121.3, 120.0, 119.6, 118.6, 118.4, 114, 112.8, 112.6, 111.4, 111.2, 109.5, 54.3; HR-EIMS: m/z calcd for C_31_H_22_N_6_O_2_S [M]^+^ 542.1525, Found 542.1515.

#### 4-((5-(4-(Di(1*H*-indol-3-yl)methyl)phenyl)-1,3,4-thiadiazol-2-yl)amino)benzene-1,2-diol (**4**)

Yield 89%, ^1^H-NMR (500 MHz, DMSO-*d*_*6*_): *δ* 10.90 (s, 2H, NH), 10.30 (s, 1H, NH), 10.10 (s, 1H, OH), 9.40 (s, 1H, OH) 7.75 (d, *J* = 7.5 Hz, 2H, Ar), 7.56–7.50 (m, 4H, Ar), 7.26 (d, *J* = 7.0 Hz, 2H, Ar), 6.92 (d, *J* = 6.8 Hz, 1H, Ar), 6.81 (dd, *J* = 8.2, 2.5 Hz, 2H, Ar), 6.70 (dd, *J* = 7.9, 3.2 Hz, 2H, Ar), 6.63 (d, *J* = 7.0 Hz, 2H, Ar), 6.61 (d, *J* = 6.5 Hz, 1H, Ar), 6.54 (s, 1H, Ar), 6.24 (s, 1H, CH),^13^C-NMR (125 MHz, DMSO-*d*_*6*_): *δ* 174.3, 152.6, 148.6, 138.7, 137.3, 136.8, 136.4, 136.2, 130.3, 129.7, 129.3, 127.8, 127.6, 127.4, 127.2, 123.5, 123.3, 121.5, 121.3, 119.5, 119.2, 118.6, 118.3, 118.2, 114.5, 112.6, 112.4, 111.4, 111.2, 102.2, 54.3; HR-EIMS: m/z calcd for C_31_H_23_N_5_O_2_S [M]^+^ 529.1572, Found 529.1561.

#### 5-(4-(Di(1*H*-indol-3-yl)methyl)phenyl)-*N*-(p-tolyl)-1,3,4-thiadiazol-2-amine (**5**)

Yield 83%, ^1^H-NMR (500 MHz, DMSO-*d*_*6*_): *δ* 11.65 (s, 2H, NH), 10.85 (s, 1H, NH), 7.74 (d, *J* = 7.5 Hz, 2H, Ar), 7.53 (d, *J* = 7.3 Hz, 2H, Ar), 7.31 (d, *J* = 7.2 Hz, 2H, Ar), 7.21 (d, *J* = 7.1 Hz, 2H, Ar), 7.20 (d, *J* = 7.0 Hz, 2H, Ar), 6.81–677 (m, 4H, Ar), 6.70 (dd, *J* = 7.9, 3.2 Hz, 2H, Ar), 6.63 (d, *J* = 7.0 Hz, 2H, Ar), 6.31 (s, 1H, CH), 2.30 (s, 3H, CH_3_); ^13^C-NMR (125 MHz, DMSO-*d*_*6*_): *δ* 174.3, 152.2, 138.2, 137.3, 136.2, 136.2, 131.5, 130.3, 129.5, 129.4, 129.2, 129.2, 127.1, 127.1, 127.0, 126.1, 123.4, 123.1, 121.5, 121.3, 120.5, 120.2, 119.6, 119.3, 118.6, 118.3, 112.4, 112.0, 111.6, 111.3, 54.4, 21.1, HR-EIMS: m/z calcd for C_32_H_25_N_5_S [M]^+^ 511.1831, Found 511.1816.

#### 5-(4-(Di(1*H*-indol-3-yl)methyl)phenyl)-*N*-(o-tolyl)-1,3,4-thiadiazol-2-amine (**6**)

Yield 84%, ^1^H-NMR (500 MHz, DMSO-*d*_*6*_): *δ* 10.50 (s, 2H, NH), 9.71 (s, 1H, NH), 7.75 (d, *J* = 7.5 Hz, 2H, Ar), 7.48 (d, *J* = 7.3 Hz, 2H, Ar), 7.20 (d, *J* = 7.0 Hz, 2H, Ar), 7.17 (dd, *J* = 8.1 3.2 Hz, 1H, Ar), 7.12 (d, *J* = 7.1 Hz, 1H, Ar), 7.10 (d, *J* = 6.9 Hz, 1H, Ar), 6.92 (dd, *J* = 7.8 2.5 Hz, 1H, Ar), 6.82–678 (m, 4H, Ar), 6.70 (dd, *J* = 7.9, 3.2 Hz, 2H, Ar), 6.57 (d, *J* = 7.0 Hz, 2H, Ar), 6.20 (s, 1H, CH), 2.10 (s, 1H, CH_3_); ^13^C-NMR (125 MHz, DMSO-*d*_*6*_): *δ* 174.4, 152.4, 142.3, 138.5, 136.2, 136.3, 131.3, 130.1, 129.8, 129.4, 129.0, 127.8, 127.6, 127.4, 127.2, 126.3, 123.8, 123.5, 123.3, 123.1, 121.4, 121.1, 119.6, 119.4, 118.4, 118.2, 112.4, 112.2, 111.5, 111.3, 54.3, 17.3; HR-EIMS: m/z calcd for C_32_H_25_N_5_S [M]^+^ 511.1831, Found 511.1816.

#### 2-((5-(4-(Di(1*H*-indol-3-yl)methyl)phenyl)-1,3,4-thiadiazol-2-yl)amino)benzene-1,4-diol (**7**)

Yield 92%, ^1^H-NMR (500 MHz, DMSO-*d*_*6*_): *δ* 11.91 (s, 2H, NH), 10.30 (s, 1H, NH), 8.90 (s, 2H, OH), 7.75 (d, *J* = 7.5 Hz, 2H, Ar), 7.54 (d, *J* = 7.3 Hz, 2H, Ar), 7.24 (d, *J* = 7.0 Hz, 2H, Ar), 6.80–6.75 (m, 4H, Ar), 6.70 (dd, *J* = 7.9, 3.2 Hz, 2H, Ar), 6.56 (d, *J* = 6.4 Hz, 2H, Ar), 6.51 (d, *J* = 7.0 Hz, 2H, Ar), 6.49 (s, 1H), 6.22 (s, 1H, CH); ^13^C-NMR (125 MHz, DMSO-*d*_*6*_): *δ* 174.6, 152.4, 151.2, 138.2, 137.3, 136.8, 136.6, 135.3, 130.2, 129.7, 129.3, 127.8, 127.6, 127.4, 127.2, 123.4, 123.2, 121.3, 121.0, 120.2, 119.0, 118.6, 118.4, 112.4, 112.2, 111.5, 111.3, 111.0, 107.0, 102.2, 54.3; HR-EIMS: m/z calcd for C_31_H_23_N_5_O_2_S [M]^+^ 529.1572, Found 529.1561.

#### 3-((5-(4-(Di(1*H*-indol-3-yl)methyl)phenyl)-1,3,4-thiadiazol-2-yl)amino)benzene-1,2-diol (**8**)

Yield 81%, ^1^H-NMR (500 MHz, DMSO-*d*_*6*_): *δ* 10.50 (s, 2H, NH), 10.32 (s, 2H, OH), 9.76 (s, 1H, NH), 7.74 (d, *J* = 7.5 Hz, 2H, Ar), 7.50 (d, *J* = 7.3 Hz, 2H, Ar), 7.20 (d, *J* = 7.0 Hz, 2H, Ar), 6.84–6.78 (m, 4H, Ar), 6.70 (dd, *J* = 7.9, 3.2 Hz, 2H, Ar), 6.58 (d, *J* = 7.0 Hz, 2H, Ar), 6.57 (dd, *J* = 7.1 2.5 Hz, 1H, Ar), 6.55 (d, *J* = 6.8 Hz, 1H, Ar), 6.50 (d, *J* = 6.7 Hz, 1H, Ar), 6.32 (s, 1H, CH); ^13^C-NMR (125 MHz, DMSO-*d*_*6*_): δ 174.0, 152.5, 148.3, 138.0, 136.8, 136.2, 135.3, 133.4, 130.2, 129.4, 129.0, 127.8, 127.5, 127.2, 127.1, 123.2, 123.0, 122.4, 121.5, 121.2, 119.4, 119.2, 118.5, 118.1, 112.4, 112.0, 111.4, 111.2, 107.1, 105.2, 54.2; HR-EIMS: m/z calcd for C_31_H_23_N_5_O_2_S [M]^+^ 529.1572, Found 529.1561.

#### 5-(4-(Di(1*H*-indol-3-yl)methyl)phenyl)-*N*-(4-fluorophenyl)-1,3,4-thiadiazol-2-amine (**9**)

Yield 90%, ^1^H-NMR (500 MHz, DMSO-*d*_*6*_): *δ* 12.18 (s, 2H, NH), 11.48 (s, 1H, NH), 7.75 (d, *J* = 7.5 Hz, 2H, Ar), 7.53 (d, *J* = 7.3 Hz, 2H, Ar), 7.40 (d, *J* = 7.4 Hz, 2H, Ar), 7.30 (d, *J* = 7.1 Hz, 2H, Ar), 7.20 (d, *J* = 7.0 Hz, 2H, Ar), 6.83–6.79 (m, 4H, Ar), 6.70 (dd, *J* = 7.9, 3.2 Hz, 2H, Ar), 6.63 (d, *J* = 7.0 Hz, 2H, Ar), 6.22 (s, 1H, CH); ^13^C-NMR (125 MHz, DMSO-*d*_*6*_): *δ* 174.3, 157.1, 152.3, 138.3, 136.7, 136.3, 136.0, 130.2, 129.3, 129.1, 127.8, 127.6, 127.3, 127.1, 123.3, 123.1, 121.5, 121.2, 120.4, 120.2, 119.4, 119.1, 118.4, 118.2, 116.5, 116.1, 112.6, 112.4, 111.3, 111.0, 54.4; HR-EIMS: m/z calcd for C_31_H_22_FN_5_S [M]^+^ 515.1580, Found 515.1566.

#### 5-(4-(Di(1*H*-indol-3-yl)methyl)phenyl)-*N*-(pyridin-3-yl)-1,3,4-thiadiazol-2-amine (**10**)

Yield 83%, ^1^H-NMR (500 MHz, DMSO-*d*_*6*_): *δ* 11.60 (s, 2H, NH), 9.20 (s, 1H, NH), 8.02 (s, 1H, Ar), 7.90 (d, *J* = 7.5 Hz, 1H, Ar), 7.74 (d, *J* = 7.5 Hz, 2H, Ar), 7.48 (d, *J* = 7.3 Hz, 2H, Ar), 7.33 (dd, *J* = 8.1 2.4 Hz, 1H, Ar), 7.20 (d, *J* = 7.0 Hz, 2H, Ar), 7.12 (d, *J* = 7.2 Hz, 1H, Ar), 6.80 (dd, *J* = 8.2, 2.5 Hz, 2H, Ar), 6.70 (dd, *J* = 7.9, 3.2 Hz, 2H, Ar), 6.63–655 (m, 4H, Ar), 6.22 (s, 1H, CH); ^13^C-NMR (125 MHz, DMSO-*d*_*6*_): *δ* 174.4, 152.4, 138.6, 138.3, 137.3, 136.2, 136.0, 133.5, 130.3, 129.2, 129.1, 127.7, 127.5, 127.3, 127.2, 124.0, 123.3, 123.1, 122.4, 121.4, 121.2, 119.5, 119.2, 118.4, 118.2, 112.4, 112.2, 111.5, 111.3, 54.3; HR-EIMS: m/z calcd for C_30_H_22_N_6_S [M]^+^ 498.1627, Found 498.1612.

#### 2-((5-(4-(Di(1*H*-indol-3-yl)methyl)phenyl)-1,3,4-thiadiazol-2-yl)amino)-5-methoxyphenol (**11**)

Yield 83%, ^1^H-NMR (500 MHz, DMSO-*d*_*6*_): *δ* 10.50 (s, 2H, NH), 9.77 (s, 1H, NH), 10.03 (s, 1H, OH), 7.74 (d, *J* = 7.5 Hz, 2H, Ar), 7.50 (d, *J* = 7.3 Hz, 2H, Ar), 7.20 (d, *J* = 7.0 Hz, 2H, Ar), 7.11 (d, *J* = 7.3 Hz, 1H, Ar), 6.83 (dd, *J* = 8.2, 2.5 Hz, 2H, Ar), 6.78 (s, 1H, Ar), 6.70–6.64 (m, 4H, Ar), 6.67 (d, *J* = 7.0 Hz, 1H, Ar), 6.62 (d, *J* = 7.0 Hz, 2H, Ar), 6.22 (s, 1H, CH), 3.72 (s, 3H, CH_3_); ^13^C-NMR (125 MHz, DMSO-*d*_*6*_): *δ* 174.3, 152.5, 150.3, 138.0, 136.4, 136.2, 134.1, 130.4, 130.2, 129.4, 129.1, 127.7, 127.5, 127.3, 127.1, 123.4, 123.2, 121.7, 121.5, 121.1, 119.4, 119.1, 118.4, 118.2, 117.1, 112.5, 112.3, 112.0, 111.4, 111.2, 55.4, 54.2, 17.5; HR-EIMS: m/z calcd for C_32_H_25_N_5_O_2_S [M]^+^ 543.1729, Found 543.1717.

#### 2-((5-(4-(Di(1*H*-indol-3-yl)methyl)phenyl)-1,3,4-thiadiazol-2-yl)amino)phenol (**12**)

Yield 81%, ^1^H-NMR (500 MHz, DMSO-*d*_*6*_): *δ* 11.50 (s, 2H, NH), 9.77 (s, 1H, NH), 9.93 (s, 1H, OH), 7.74 (d, *J* = 7.5 Hz, 2H, Ar), 7.50 (d, *J* = 7.3 Hz, 2H, Ar), 7.20 (d, *J* = 7.0 Hz, 2H, Ar), 7.10 (dd, *J* = 8.3, 3.3 Hz, 1H, Ar), 7.00 (d, *J* = 7.1 Hz, 1H, Ar), 6.82 (dd, *J* = 7.8, 3.5 Hz, 1H, Ar), 6.81 (d, *J* = 7.1 Hz, 1H, Ar), 6.80 (dd, *J* = 8.2, 2.5 Hz, 2H, Ar), 6.70–6.65 (m, 4H, Ar), 6.60 (d, *J* = 7.0 Hz, 2H, Ar), 6.20 (s, 1H, CH); ^13^C-NMR (125 MHz, DMSO-*d*_*6*_): *δ* 174.0, 152.5, 144.2, 138.0, 136.3, 136.1, 134.0, 130.2, 129.3, 129.4, 127.2, 127.1, 126.8, 126.4, 122.9, 122.7, 122.0, 121.5, 121.3, 120.0, 119.6, 119.4, 118.5, 118.3, 116.4, 112.2, 112.0, 111.9, 111.4, 111.1, 54.4; HR-EIMS: m/z calcd for C_31_H_23_N_5_OS [M]^+^ 513.1623, Found 513.1609.

#### 5-(4-(Di(1*H*-indol-3-yl)methyl)phenyl)-*N*-(4-nitrophenyl)-1,3,4-thiadiazol-2-amine (**13**)

Yield 88%, ^1^H-NMR (500 MHz, DMSO-*d*_*6*_): *δ* 12.60 (s, 2H, NH), 12.24 (s, 1H, NH), 8.00 (d, *J* = 7.7 Hz, 2H, Ar), 7.74 (d, *J* = 7.5 Hz, 2H, Ar), 7.54 (d, *J* = 7.3 Hz, 2H, Ar), 7.42 (d, *J* = 7.2 Hz, 2H, Ar), 7.20 (d, *J* = 7.0 Hz, 2H, Ar), 6.82 (dd, *J* = 8.2, 2.5 Hz, 2H, Ar), 6.70 (dd, *J* = 7.9, 3.2 Hz, 2H, Ar), 6.57–652 (d, *J* = 7.0 Hz, 4H, Ar), 6.22 (s, 1H, CH); ^13^C-NMR (125 MHz, DMSO-*d*_*6*_): *δ* 174.0, 152.4, 146.4, 138.0, 137.7, 136.8, 136.3, 130.4, 129.7, 129.3, 127.6, 127.3, 127.9, 127.6, 124.4, 124.2, 123.4, 123.1, 121.5, 121.2, 119.6, 119.4, 119.2, 119.0, 118.6, 118.3, 112.6, 112.3, 111.5, 111.2, 54.3; HR-EIMS: m/z calcd for C_31_H_22_N_6_O_2_S [M]^+^ 542.1525, Found 542.1515.

#### 5-((5-(4-(Di(1*H*-indol-3-yl)methyl)phenyl)-1,3,4-thiadiazol-2-yl)amino)benzene-1,3-diol (**14**)

Yield 77%, ^1^H-NMR (500 MHz, DMSO-*d*_*6*_): *δ* 12.60 (s, 2H, NH), 10.30 (s, 1H, NH), 9.33 (s, 2H, OH), 7.75 (d, *J* = 7.5 Hz, 2H, Ar), 7.50 (d, *J* = 7.3 Hz, 2H, Ar), 7.20–716 (m, 5H, Ar), 6.83 (dd, *J* = 8.2, 2.5 Hz, 2H, Ar), 6.68–6.64 (m, 4H, Ar), 6.58 (d, *J* = 7.0 Hz, 2H, Ar), 6.23 (s, 1H, CH); ^13^C-NMR (125 MHz, DMSO-*d*_*6*_): *δ* 174.0, 160.5, 160.5, 152.8, 145.0, 138.0, 136.3, 136.1, 130.7, 129.8, 129.3, 127.9, 127.7, 127.5, 127.2, 123.5, 123.1, 121.6, 121.2, 119.4, 119.0, 118.8, 118.2, 113.6, 112.3, 111.8, 111.4, 95.6, 95.3, 93.4, 54.2; HR-EIMS: m/z calcd for C_31_H_23_N_5_O_2_S [M]^+^ 529.1572, Found 529.1561.

#### 4-((5-(4-(Di(1*H*-indol-3-yl)methyl)phenyl)-1,3,4-thiadiazol-2-yl)amino)phenol (**15**)

Yield 90%, ^1^H-NMR (500 MHz, DMSO-*d*_*6*_): *δ* 11.60 (s, 2H, NH), 10.30 (s, 1H, NH), 9.58 (s, 1H, OH), 7.75 (d, *J* = 7.5 Hz, 2H, Ar), 7.61 (d, *J* = 7.0 Hz, 2H, Ar), 7.55 (d, *J* = 7.3 Hz, 2H, Ar), 7.20 (d, *J* = 7.0 Hz, 2H, Ar), 6.94 (d, *J* = 6.7 Hz, 2H, Ar), 6.84 (dd, *J* = 8.2, 2.5 Hz, 2H, Ar), 6.70–6.65 (m, 4H, Ar), 6.64 (d, *J* = 7.0 Hz, 2H, Ar), 6.22 (s, 1H, CH); ^13^C-NMR (125 MHz, DMSO-*d*_*6*_): *δ* 174.7, 174.4, 158.3, 137.9, 136.8, 136.3, 130.2, 129.7, 129.3, 128.9, 128.3, 127.1, 127.0, 126.7, 126.4, 126.2, 123.5, 123.1, 121.6, 121.4, 119.5, 119.2, 118.6, 118.2, 116.2, 116.1, 112.5, 112.2, 111.7, 111.2, 54.3; HR-EIMS: m/z calcd for C_31_H_23_N_5_OS [M]^+^ 513.1623, Found 513.1609.

#### 5-(4-(Di(1*H*-indol-3-yl)methyl)phenyl)-*N*-(pyridin-2-yl)-1,3,4-thiadiazol-2-amine (**16**)

Yield 79%, ^1^H-NMR (500 MHz, DMSO-*d*_*6*_): *δ* 12.70 (s, 2H, NH), 10.30 (s, 1H, NH), 8.56 (d, *J* = 8.1 Hz, 1H, Ar), 8.08 (d, *J* = 7.8 Hz, 1H, Ar), 7.82 (dd, *J* = 8.1, 3.5 Hz, 1H, Ar), 7.74 (d, *J* = 7.5 Hz, 2H, Ar), 7.50 (d, *J* = 7.3 Hz, 2H, Ar), 7.37 (dd, *J* = 7.3 Hz, 1H, Ar), 7.20 (d, *J* = 7.0 Hz, 2H, Ar), 6.82 (dd, *J* = 8.2, 2.5 Hz, 2H, Ar), 6.70 (dd, *J* = 7.9, 3.2 Hz, 2H, Ar), 6.63–6.59 (m, 4H, Ar), 6.19 (s, 1H, CH); ^13^C-NMR (125 MHz, DMSO-*d*_*6*_): *δ* 174.4, 174.1, 157.0, 149.8, 138.3, 137.8, 136.7, 136.6, 130.4, 129.3, 129.1, 127.9, 127.6, 127.4, 127.1, 124.5, 123.3, 123.1, 122.8, 121.4, 121.2, 119.6, 119.4, 118.5, 118.2, 112.4, 112.0, 111.3, 111.2, 54.4; HR-EIMS: m/z calcd for C_30_H_22_N_6_S [M]^+^ 498.1627, Found 498.1612.

#### 5-(4-(Di(1*H*-indol-3-yl)methyl)phenyl)-*N*-(2-fluorophenyl)-1,3,4-thiadiazol-2-amine (**17**)

Yield 71%, ^1^H-NMR (500 MHz, DMSO-*d*_*6*_): *δ* 12.63 (s, 2H, NH), 11.81 (s, 1H, NH), 8.38 (s, 1H, Ar), 7.74 (d, *J* = 7.5 Hz, 2H, Ar), 7.71 (d, *J* = 7.5 Hz, 1H, Ar), 7.70 (dd, *J* = 7.9, 3, 5 Hz, 1H, Ar), 7.50 (d, *J* = 7.3 Hz, 2H, Ar), 7.46 (d, *J* = 7.7 Hz, 1H, Ar), 7.25 (dd, *J* = 7.4 Hz, 1H, Ar), 7.20 (d, *J* = 7.0 Hz, 2H, Ar), 6.83 (dd, *J* = 8.2, 2.5 Hz, 2H, Ar), 6.70 (dd, *J* = 7.9, 3.2 Hz, 2H, Ar), 6.63–6.58 (m, 3H, Ar), 6.22 (s, 1H, CH); ^13^C-NMR (125 MHz, DMSO-*d*_*6*_): *δ* 174.4, 174.2, 158.1, 138.5, 136.7, 136.2, 130.4, 130.1, 129.9, 129.5, 129.3, 127.5, 127.4, 127.2, 127.0, 125.1, 123.6, 123.1, 122.6, 121.5, 121.0, 119.4, 119.0, 118.2, 118.1, 114.5, 112.0, 111.8, 111.4, 110.4, 54.3; HR-EIMS: m/z calcd for C_31_H_22_FN_5_S [M]^+^ 515.1580, Found 515.1566.

#### 5-((5-(4-(Di(1*H*-indol-3-yl)methyl)phenyl)-1,3,4-thiadiazol-2-yl)amino)-2-methoxyphenol (**18**)

Yield 78%, ^1^H-NMR (500 MHz, DMSO-*d*_*6*_): *δ* 12.10 (s, 2H, NH), 10.30 (s, 1H, NH), 10.08 (s, 1H, OH), 7.73 (d, *J* = 7.5 Hz, 2H, Ar), 7.50 (d, *J* = 7.3 Hz, 2H, Ar), 7.30 (d, *J* = 7.3 Hz, 1H, Ar), 7.20 (d, *J* = 7.0 Hz, 2H, Ar), 7.12 (s, 1H, Ar), 6.83 (dd, *J* = 8.2, 2.5 Hz, 2H, Ar), 6.80 (d, *J* = 6.8 Hz, 1H, Ar), 6.70 (dd, *J* = 7.9, 3.2 Hz, 2H, Ar), 6.63–6.58 (m, 4H, Ar), 6.23 (s, 1H, CH), 3.84 (s, 3H, CH_3_); ^13^C-NMR (125 MHz, DMSO-*d*_*6*_): *δ* 174.6, 174.3, 147.9, 147.7, 138.0, 136.4, 136.2, 130.9, 129.2, 129.0, 127.9, 127.7, 127.6, 127.3, 127.0, 123.6, 123.3, 121.8, 121.5, 121.2, 119.6, 119.3, 118.6, 118.2, 113.6, 112.5, 112.2, 111.2, 111.7, 110.0, 56.0, 54.3; HR-EIMS: m/z calcd for C_32_H_25_N_5_O_2_S [M]^+^ 543.1729, Found 543.1717.

#### *N*-(3-Chlorophenyl)-5-(4-(di(1*H*-indol-3-yl)methyl)phenyl)-1,3,4-thiadiazol-2-amine (**19**)

Yield 70%, ^1^H-NMR (500 MHz, DMSO-*d*_*6*_): *δ* 12.50 (s, 2H, NH), 11.92 (s, 1H, NH), 8.32 (s, 2H, Ar), 7.94 (s, 1H, Ar), 7.90 (d, *J* = 7.7 Hz, 1H, Ar), 7.74 (d, *J* = 7.5 Hz, 2H, Ar), 7.50 (d, *J* = 7.3 Hz, 2H, Ar), 7.45 (d, *J* = 7.5 Hz, 1H, Ar), 7.46 (d, *J* = 7.3 Hz, 1H, Ar), 7.20 (d, *J* = 7.0 Hz, 2H, Ar), 6.83 (dd, *J* = 8.2, 2.5 Hz, 2H, Ar), 6.70 (dd, *J* = 7.9, 3.2 Hz, 2H, Ar), 6.64 (d, *J* = 7.0 Hz, 2H, Ar), 6.12 (s, 1H, CH); ^13^C-NMR (125 MHz, DMSO-*d*_*6*_): *δ* 174.6, 174.2, 138.4, 136.2, 136.0, 134.7, 134.3, 130.2, 129.9, 129.7, 129.4, 129.2, 128.5, 127.8, 127.5, 127.2, 127.0, 126.4, 124.0, 123.6, 122.7, 121.4, 120.8, 119.3, 118.3, 118.0, 113.1, 112.4, 112.8, 111.4, 54.3; HR-EIMS: m/z calcd for C_31_H_22_ClN_5_S [M]^+^ 531.1284, Found 531.1270.

#### 2-((5-(4-(Di(1*H*-indol-3-yl)methyl)phenyl)-1,3,4-thiadiazol-2-yl)amino)-4-methoxyphenol (**20**)

Yield 81%, ^1^H-NMR (500 MHz, DMSO-*d*_*6*_): *δ* 12.20 (s, 2H, NH), 10.64 (s, 1H, NH), 10.25 (s, 1H, OH), 7.73 (d, *J* = 7.5 Hz, 2H, Ar), 7.55 (d, *J* = 7.3 Hz, 2H, Ar), 7.20 (d, *J* = 7.0 Hz, 2H, Ar), 7.17 (s, 1H, Ar), 6.81 (dd, *J* = 8.2, 2.5 Hz, 2H, Ar), 6.70 (dd, *J* = 7.9, 3.2 Hz, 2H, Ar), 6.68 (d, *J* = 6.8 Hz, 1H, Ar), 6.66 (d, *J* = 6.7 Hz, 1H, Ar), 6.61–6.56 (m, 4H, Ar), 6.12 (s, 1H, CH), 3.84 (s, 3H, CH_3_); ^13^C-NMR (125 MHz, DMSO-*d*_*6*_): *δ* 174.7, 174.3, 153.5, 147.2, 138.0, 137.5, 136.3, 130.2, 129.1, 128.5, 128.8, 127.6, 127.3, 127.1, 123.7, 123.5, 123.1, 122.7, 122.3, 119.4, 119.2, 118.6, 118.1, 117.2, 115.3, 113.7, 113.1, 112.5, 111.6, 111.4, 55.5, 54.3; HR-EIMS: m/z calcd for C_32_H_25_N_5_O_2_S [M]^+^ 543.1729, Found 543.1717.

#### 5-(4-(Di(1*H*-indol-3-yl)methyl)phenyl)-*N*-(2-nitrophenyl)-1,3,4-thiadiazol-2-amine (**21**)

Yield 83%, ^1^H-NMR (500 MHz, DMSO-*d*_*6*_): *δ* 12.75 (s, 2H, NH), 12.25 (s, 1H, NH), 8.16 (d, *J* = 8.0 Hz, 1H, Ar), 8.04 (d, *J* = 7.9 Hz, 1H, Ar), 7.85 (dd, *J* = 8.0, 3.4 Hz, 1H, Ar), 7.75 (d, *J* = 7.5 Hz, 2H, Ar), 7.70 (d, *J* = 7.1 Hz, 1H, Ar), 7.50 (d, *J* = 7.3 Hz, 2H, Ar), 7.20 (d, *J* = 7.0 Hz, 2H, Ar), 6.82 (dd, *J* = 8.2, 2.5 Hz, 2H, Ar), 6.70 (dd, *J* = 7.9, 3.2 Hz, 2H, Ar), 6.64–659 (m, 4H, Ar), 6.22 (s, 1H, CH); ^13^C-NMR (125 MHz, DMSO-*d*_*6*_): *δ* 175.1, 174.0, 147.4, 139.1, 137.2, 136.2, 135.0, 131.2, 130.8, 129.4, 129.2, 128.5, 127.4, 127.2, 126.8, 126.4, 126.0, 124.8, 123.6, 123.3, 121.5, 121.2, 119.5, 119.1, 118.4, 118.0, 113.1, 112.7, 111.6, 111.3, 54.3; HR-EIMS: m/z calcd for C_31_H_22_N_6_O_2_S [M]^+^ 542.1525, Found 542.1515.

#### 4-((5-(4-(Di(1*H*-indol-3-yl)methyl)phenyl)-1,3,4-thiadiazol-2-yl)amino)benzene-1,3-diol (**22**)

Yield 83%, ^1^H-NMR (500 MHz, DMSO-*d*_*6*_): *δ* 12.40 (s, 2H, NH), 11.20 (s, 1H, NH), 10.40 (s, 2H, OH), 7.76 (d, *J* = 7.0 Hz, 2H, Ar), 7.52 (d, *J* = 7.5 Hz, 2H, Ar), 7.22 (d, *J* = 7.0 Hz, 2H, Ar), 7.13 (d, *J* = 7.5 Hz, 1H, Ar), 6.80 (d, *J* = 8.0 Hz, 2H, Ar), 6.78 (s, 1H, Ar), 6.70 (dd, *J* = 7.9, 3.2 Hz, 2H, Ar), 6.64 (d, *J* = 7.5 Hz, 1H, Ar), 6.60–6.55 (m, 4H, Ar), 3.72 (s, 3H, CH_3_), 6.32 (s, 1H, CH); ^13^C-NMR (125 MHz, DMSO-*d*_*6*_): *δ* 174.6, 152.4, 150.2, 138.1, 136.5, 136.0, 134.2, 130.3, 130.2, 129.4, 129.0, 127.7, 127.5, 127.2, 127.1, 123.3, 123.2, 121.6, 121.5, 121.0, 119.3, 119.0, 118.4, 118.2, 117.1, 112.5, 112.5, 112.0, 111.4, 111.3, 55.4, 54.2; HR-EIMS: m/z calcd for C_31_H_23_N_5_O_2_S [M]^+^ 529.1572, Found 529.1561.

#### 5-(4-(Di(1*H*-indol-3-yl)methyl)phenyl)-*N*-(m-tolyl)-1,3,4-thiadiazol-2-amine (**23**)

Yield 81%, ^1^H-NMR (500 MHz, DMSO-*d*_*6*_): *δ* 12.40 (s, 2H, NH), 11.84 (s, 1H, NH), 7.75 (d, *J* = 7.5 Hz, 2H, Ar), 7.53 (d, *J* = 7.3 Hz, 1H, Ar), 7.50 (d, *J* = 7.3 Hz, 2H, Ar), 7.43 (s, 1H, Ar), 7.22 (dd, *J* = 8.4, 3.3 Hz, 1H, Ar), 7.20 (d, *J* = 7.0 Hz, 2H, Ar), 6.83 (dd, *J* = 8.2, 2.5 Hz, 2H, Ar), 6.80 (d, *J* = 7.6 Hz, 1H, Ar), 6.70 (dd, *J* = 7.9, 3.2 Hz, 2H, Ar), 6.66–6.62 (m, 2H, Ar), 6.20 (s, 1H, CH), 2.30 (s, 3H, CH_3_); ^13^C-NMR (125 MHz, DMSO-*d*_*6*_): *δ* 175.1, 152.3, 142.1, 139.0, 138.4, 136.1, 136.2, 130.1, 129.9, 129.5, 129.1, 128.4, 128.0, 127.7, 127.1, 123.8, 123.3, 121.9, 121.6, 121.4, 119.4, 119.1, 119.0, 118.6, 118.4, 114.2, 112.4, 112.2, 111.6, 111.2, 54.3, 21.0; HR-EIMS: m/z calcd for C_32_H_25_N_5_S [M]^+^ 511.1831, Found 511.1816.

#### *N*-(4-Chlorophenyl)-5-(4-(di(1*H*-indol-3-yl)methyl)phenyl)-1,3,4-thiadiazol-2-amine (**24**)

Yield 81%, ^1^H-NMR (500 MHz, DMSO-*d*_*6*_): *δ* 11.70 (s, 2H, NH), 10.92 (s, 1H, NH), 7.75 (d, *J* = 7.5 Hz, 2H, Ar), 7.63 (d, *J* = 7.6 Hz, 2H, Ar), 7.50 (d, *J* = 7.3 Hz, 2H, Ar), 7.25 (d, *J* = 7.3 Hz, 2H, Ar), 7.20 (d, *J* = 7.0 Hz, 2H, Ar), 6.81 (dd, *J* = 8.2, 2.5 Hz, 2H, Ar), 6.70 (dd, *J* = 7.9, 3.2 Hz, 2H, Ar), 6.64–6.59 (m, 4H, Ar), 6.25 (s, 1H, CH); ^13^C-NMR (125 MHz, DMSO-*d*_*6*_): *δ* 174.4, 152.2, 138.2, 138.0, 136.8, 136.2, 130.2, 129.8, 129.4, 129.2, 129.0, 127.4, 127.2, 127.0, 126.8, 126.4, 123.6, 123.3, 122.6, 122.2, 121.4, 121.1, 119.2, 119.0, 118.6, 118.2, 112.7, 112.3, 111.8, 111.4, 54.3; HR-EIMS: m/z calcd for C_31_H_22_ClN_5_S [M]^+^ 531.1284, Found 531.1270.

#### *N*-(2-Chlorophenyl)-5-(4-(di(1*H*-indol-3-yl)methyl)phenyl)-1,3,4-thiadiazol-2-amine (**25**)

Yield 93%, ^1^H-NMR (500 MHz, DMSO-*d*_*6*_): *δ* 12.80 (s, 2H, NH), 12.04 (s, 1H, NH), 8.17 (d, *J* = 7.9 Hz, 1H, Ar), 7.74 (d, *J* = 7.5 Hz, 2H, Ar), 7.54 (d, *J* = 7.5 Hz, 1H, Ar), 7.50 (d, *J* = 7.3 Hz, 2H, Ar), 7.40 (dd, *J* = 8.3, 3.3 Hz, 1H, Ar), 7.24 (dd, *J* = 7.8, 2.7 Hz, 1H, Ar), 7.20 (d, *J* = 7.0 Hz, 2H, Ar), 6.83 (dd, *J* = 8.2, 2.5 Hz, 2H, Ar), 6.72–6.68 (m, 4H, Ar), 6.63 (d, *J* = 7.0 Hz, 2H, Ar), 6.21 (s, 1H, CH); ^13^C-NMR (125 MHz, DMSO-*d*_*6*_): *δ* 174.7, 152.2, 138.0, 136.8, 136.3, 136.0, 130.4, 130.1, 129.8, 129.2, 128.6, 128.0, 127.7, 127.3, 127.0, 125.7, 123.7, 123.3, 122.5, 122.0, 121.3, 121.0, 120.4, 119.4, 118.2, 118.0, 112.6, 112.2, 111.6, 111.3, 54.3; HR-EIMS: m/z calcd for C_31_H_22_ClN_5_S [M]^+^ 531.1284, Found 531.1270.

#### 5-(4-(Di(1*H*-indol-3-yl)methyl)phenyl)-*N*-(3-fluorophenyl)-1,3,4-thiadiazol-2-amine (**26**)

Yield 88%, ^1^H-NMR (500 MHz, DMSO-*d*_*6*_): *δ* 11.70 (s, 2H, NH), 10.10 (s, 1H, NH), 7.72 (d, *J* = 7.5 Hz, 2H, Ar), 7.70 (s, 1H, Ar), 7.50 (d, *J* = 7.3 Hz, 2H, Ar), 7.42 (d, *J* = 7.5 Hz, 1H, Ar), 7.31 (dd, *J* = 8.3, 3.3 Hz, 1H, Ar), 7.20 (d, *J* = 7.0 Hz, 2H, Ar), 6.82 (dd, *J* = 8.2, 2.5 Hz, 2H, Ar), 6.73–6.68 (m, 3H, Ar), 6.70 (dd, *J* = 7.9, 3.2 Hz, 2H, Ar), 6.64 (d, *J* = 7.0 Hz, 2H, Ar), 6.22 (s, 1H, CH); ^13^C-NMR (125 MHz, DMSO-*d*_*6*_): *δ* 175.0, 163.3, 152.1, 144.4, 138.4, 136.8, 136.3, 131.4, 130.2, 129.8, 129.2, 128.4, 128.0, 127.8, 127.3, 123.5, 123.1, 121.5, 121.2, 119.4, 119.2, 118.3, 118.0, 113.1, 112.8, 112.2, 111.4, 111.0, 110.0, 104.3, 54.3; HR-EIMS: m/z calcd for C_31_H_22_FN_5_S [M]^+^ 515.1580, Found 515.1566.

#### 3-((5-(4-(Di(1*H*-indol-3-yl)methyl)phenyl)-1,3,4-thiadiazol-2-yl)amino)phenol (**27**)

Yield 90%, ^1^H-NMR (500 MHz, DMSO-*d*_*6*_): *δ* 9.90 (s, 2H, NH), 9.54 (s, 1H, NH), 8.40 (s, 1H, OH), 7.74 (d, *J* = 7.5 Hz, 2H, Ar), 7.50 (d, *J* = 7.3 Hz, 2H, Ar), 7.20 (d, *J* = 7.0 Hz, 2H, Ar), 7.18 (d, *J* = 7.5 Hz, 1H, Ar), 7.08 (d, *J* = 7.2 Hz, 1H, Ar), 6.82 (dd, *J* = 8.2, 2.5 Hz, 2H, Ar), 6.70–6.65 (m, 4H, Ar), 6.62 (s, 1H, Ar), 6.56 (d, *J* = 6.7 Hz, 1H, Ar), 6.48 (d, *J* = 7.0 Hz, 2H, Ar), 6.24 (s, 1H, CH); ^13^C-NMR (125 MHz, DMSO-*d*_*6*_): δ 174.8, 159.0, 152.3, 143.2, 138.3, 136.1, 136.0, 130.5, 130.2, 129.7, 129.3, 128.4, 126.4, 127.8, 127.2, 123.6, 123.2, 121.4, 121.1, 119.4, 119.2, 118.4, 118.0, 112.8, 112.4, 111.8, 111.1, 110.2, 109.3, 102.2, 54.1; HR-EIMS: m/z calcd for C_31_H_23_N_5_OS [M]^+^ 513.1623, Found 513.1609.

### In vitro leishmaniasis assay

The assay was carried out according to Seifert and Croft [38]. Briefly, THP-1 cells (ATCC) were cultured in RPMI-1640 (R5886 Sigma) supplemented with 1% l-glutamine and 10% HIFBS (complete medium) before harvested at 1.0 × 10^6^ cells/mL. Cells were diluted to 2.0 × 10^5^ cells/mL with the complete medium, seeded in 16-well Lab Tek tissue culture chamber slide (Fisher Scientific) at a seeding density of 5.0 × 10^4^ macrophage/well (100 μL) and allowed to adhere by the addition of PMA (Phorbol-12 myristate Acetate P8139 Sigma) for 3 days at 37 °C in a 5% CO_2_–95% air mixture. Macrophages were then infected with long-slender (stationary stage) of *Leishmania major* promastigote (JISH118) obtained from The London School of Hygiene and Tropical Medicine (LSHTM) United Kingdom, which were cultured at 26 °C in

Schneiders Drosophila medium (S0146 Sigma), at a macrophage-promastigote ratio of 1:5. Infected macrophages were maintained at 34 °C in a 5% CO_2_–95% air mixture. After 48 h, extracellular parasites were removed by substituting the overlay with new fresh RPMI-1640 medium supplemented with 1% l-glutamine. Fresh Pentamidine and test compounds with various concentrations were added and drug or compound activity was determined from the percentage of infected cells in drug-treated cultures in relation to non-treated cultures using GraphPad Prism after methanol fixation and Giemsa staining. All testing was triplicated.

## Additional file

**Additional file 1.** The file contained Proton NMR spectra.

## Data Availability

Data and materials are available.

## Abbreviations

IC_50_: the IC_50_ is the concentration of an inhibitor where the response (or binding) is reduced by half
µM: micromolar
mM: millimolar
^1^HNMR: proton nuclear magnetic resonance
^13^CNMR: ^13^carbon nuclear magnetic resonance
HR-EIMS: high-resolution electron ionization mass spectrometry
H: hours
SAR: structure activity relationship
SEM: standard error mean
Fig: figure
PTR: pteridine reductase active
Gly: glycine
Ana: asparagine
Phe: phenylalanine
Met: methionine
Leu: leucine
EIMS: electron impact mass spectra
TLC: thin layer chromatography
MTX: methotrexate
SP: standards precision
XP: extra precision
Tyr: tyrosine
