# Supplementary material for: Synthesis, anti-leishmanial and molecular docking study of bis-indole derivatives
Source: BMC Chem. 2019 Aug 6;13(1):102. doi: 10.1186/s13065-019-0617-4 (PMC6685257; doi:10.1186/s13065-019-0617-4)
Supplement: Supplementary file 1 — Additional file 1. The file contained Proton NMR spectra. [file 13065_2019_617_MOESM1_ESM.pdf]

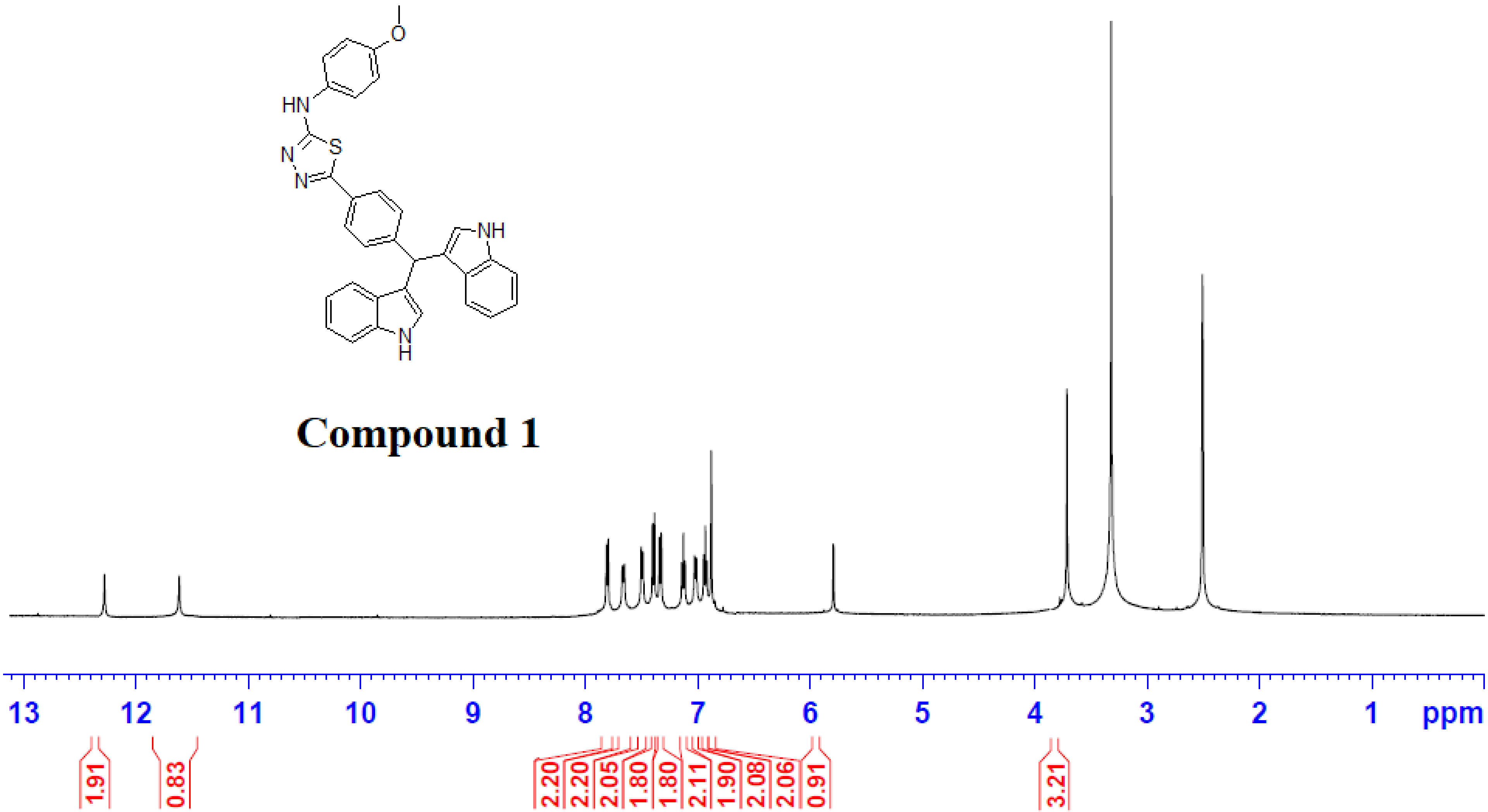

Compound 1
HN
N
N
S
O
NH
N
H
13 12 11 10 9 8 7 6 5 4 3 2 1 ppm
1.91
0.83
2.20
2.20
2.05
1.80
1.80
2.11
1.90
2.08
2.06
0.91
3.21

Compound 3

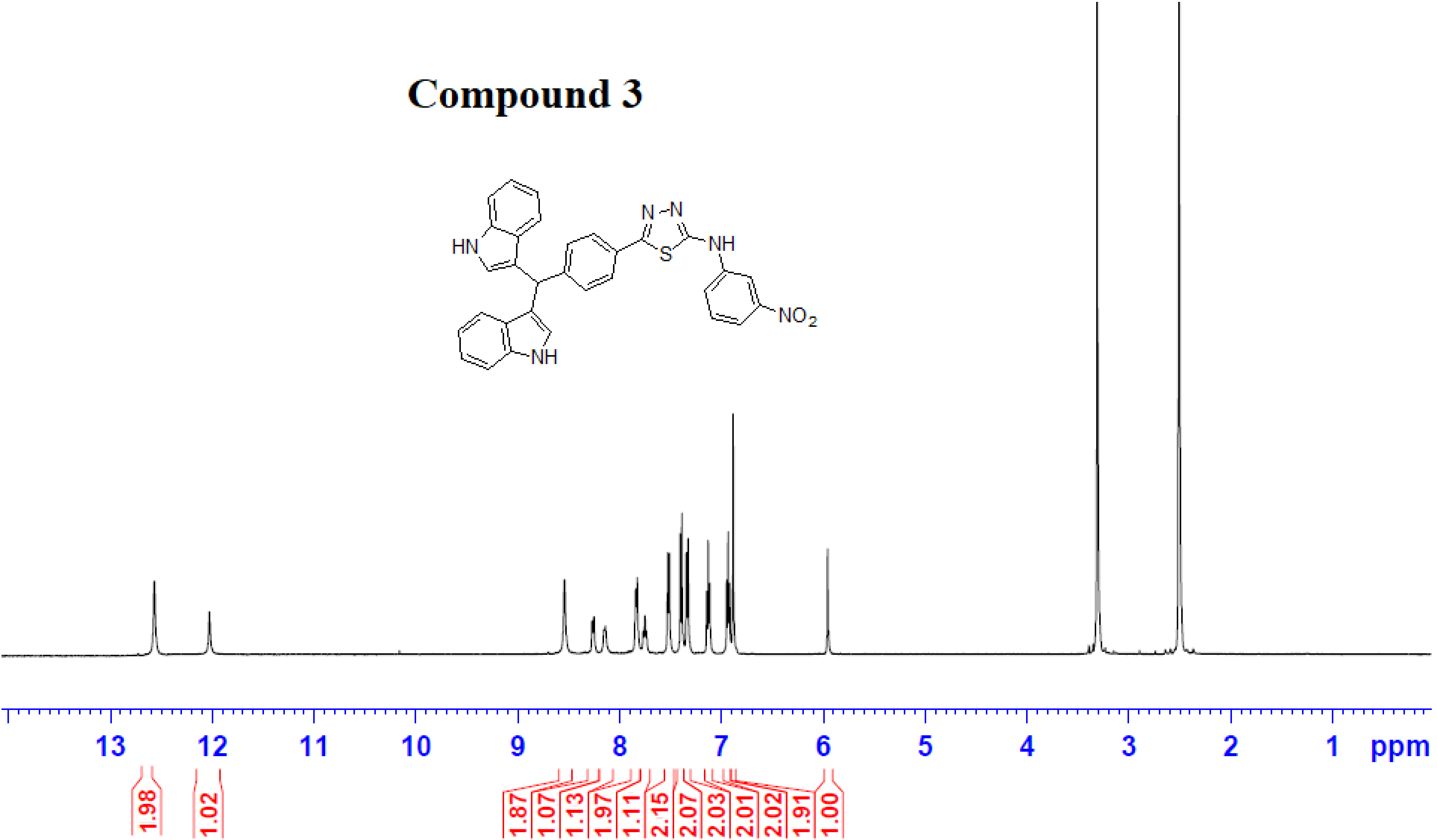

HN
N
N
S
NH
NO2
NH
13
12
11
10
9
8
7
6
5
4
3
2
1
ppm
1.98
1.02
1.87
1.07
1.13
1.97
1.11
2.15
2.07
2.03
2.01
2.02
1.91
1.00

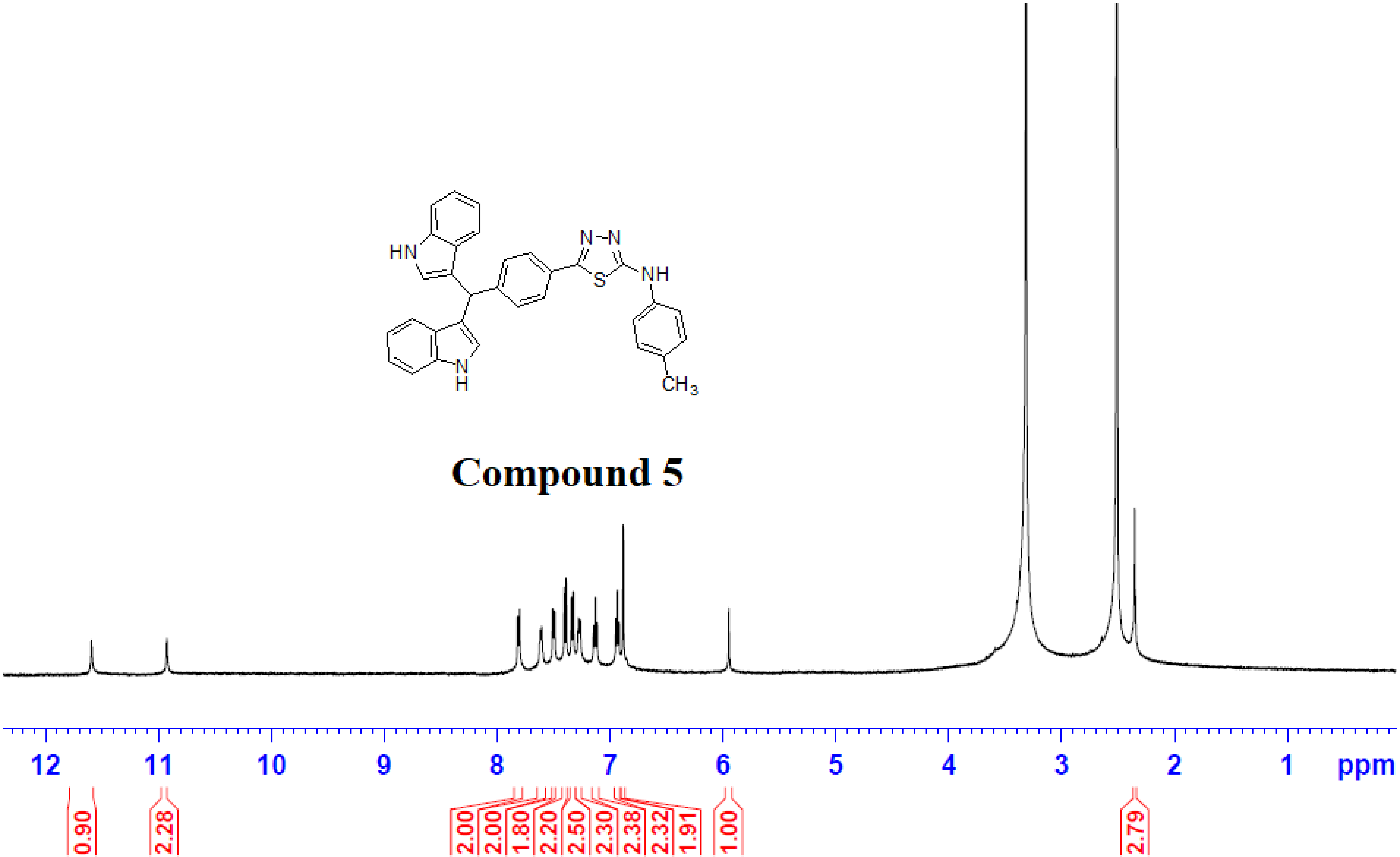
HN
N-N
S
NH
N
H
CH3
Compound 5
12
11
10
9
8
7
6
5
4
3
2
1
ppm
0.90
2.28
2.00
2.00
1.80
2.20
2.50
2.30
2.38
2.32
1.91
1.00
2.79

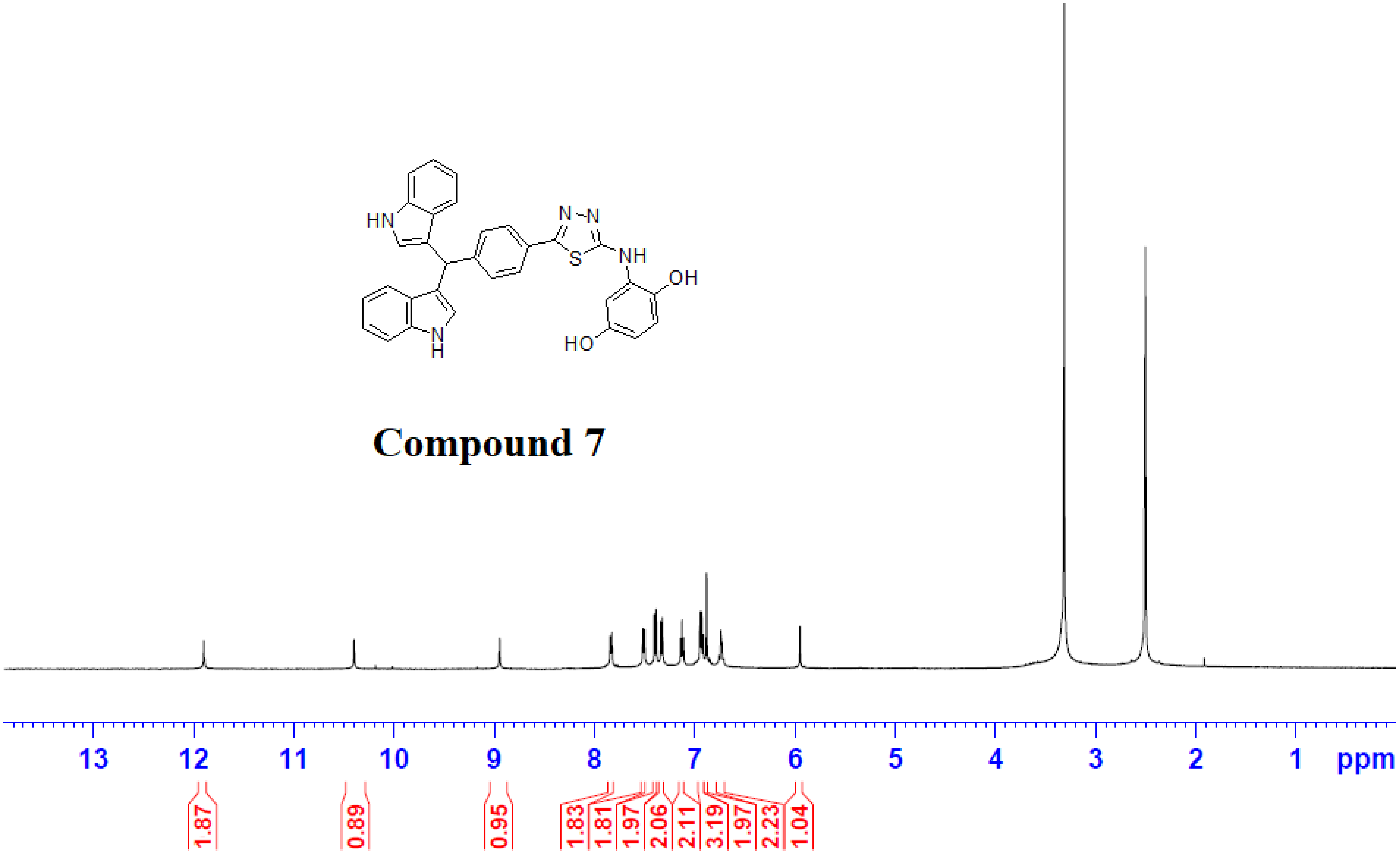
HN
N-N
S
NH
OH
HO
N
H
13
12
11
10
9
8
7
6
5
4
3
2
1
ppm
1.87
0.89
0.95
1.83
1.81
1.97
2.06
2.11
3.19
1.97
2.23
1.04

Compound 7

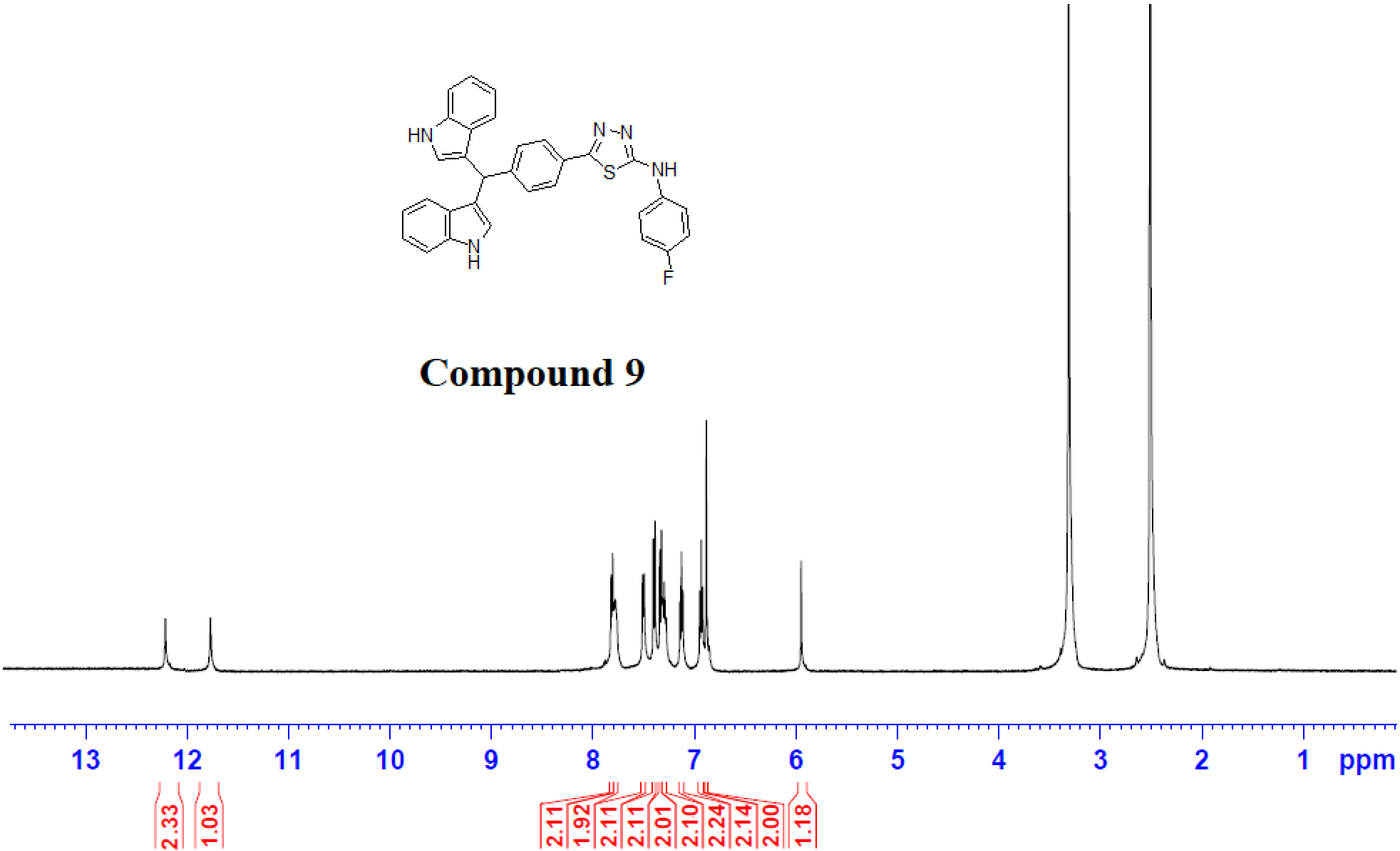
HN
N-N
NH
S
N
H
F
Compound 9
13
12
11
10
9
8
7
6
5
4
3
2
1
ppm
2.33
1.03
2.11
1.92
2.11
2.11
2.01
2.10
2.24
2.14
2.00
1.18

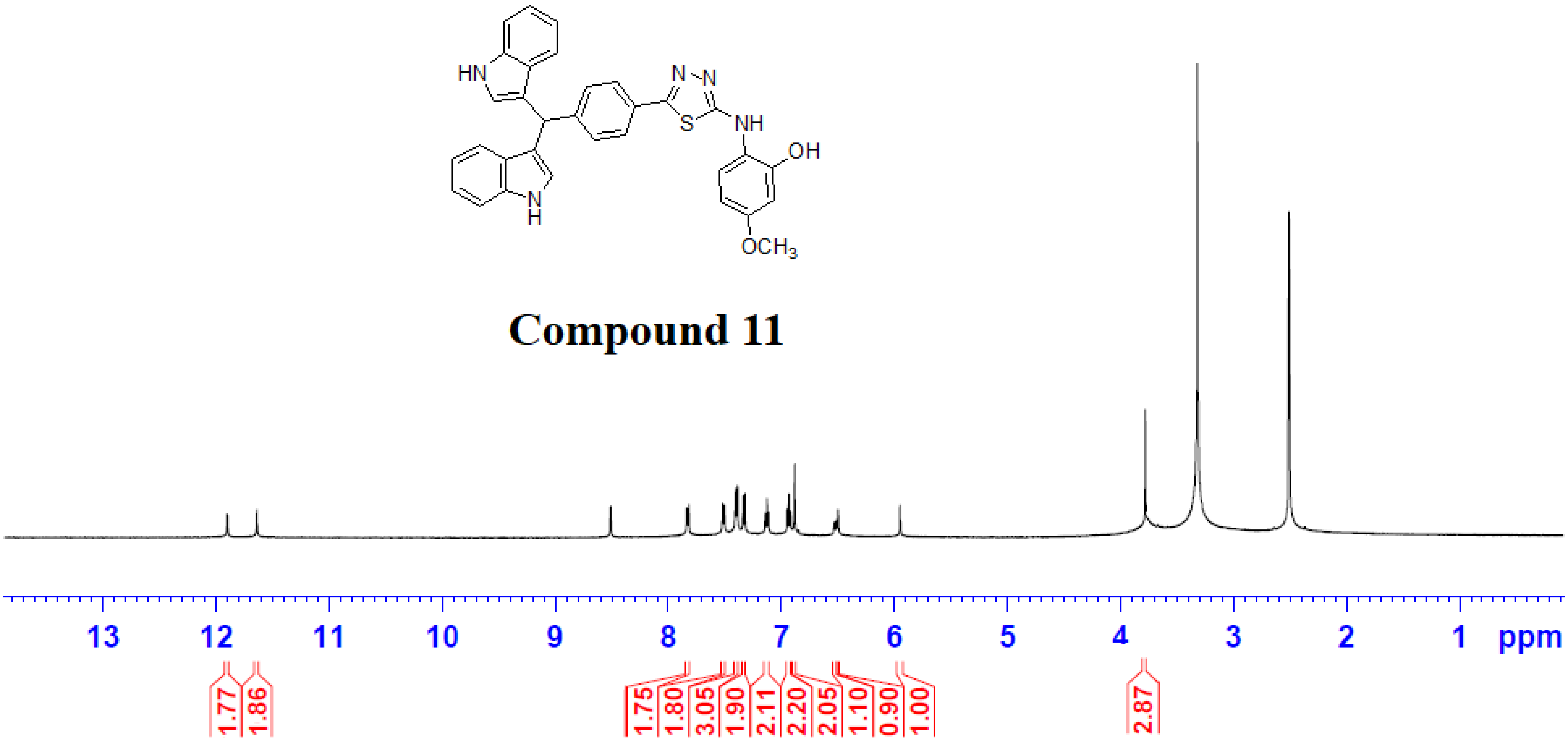

HN
N
N
S
NH
OH
N
H
OCH3
13
12
11
10
9
8
7
6
5
4
3
2
1
ppm
1.77
1.86
1.75
1.80
3.05
1.90
2.11
2.20
2.05
1.10
0.90
1.00
2.87

Compound 11

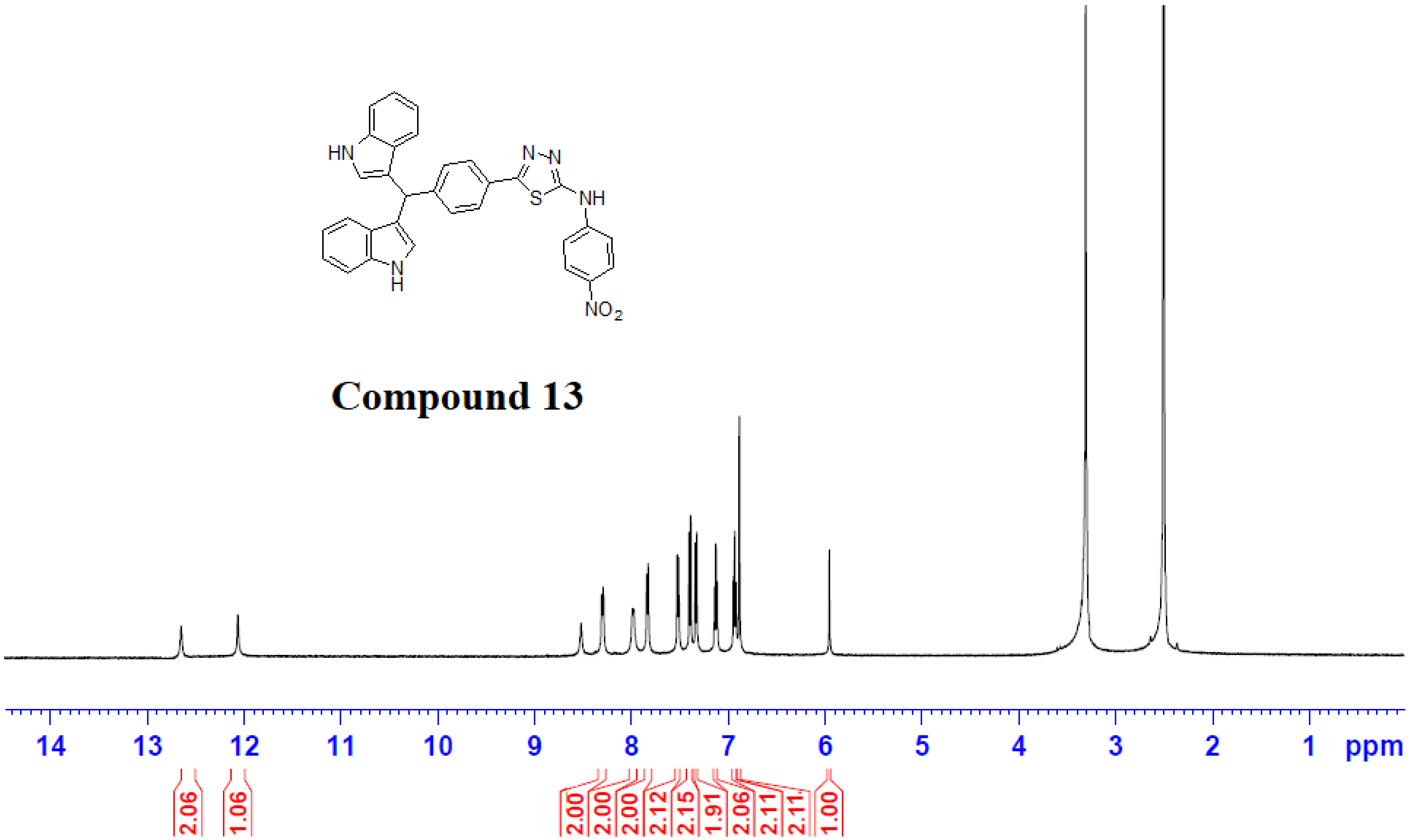
HN
N-N
S
NH
N
H
NO2
Compound 13
14 13 12 11 10 9 8 7 6 5 4 3 2 1 ppm
2.06
1.06
2.00
2.00
2.00
2.12
2.15
1.91
2.06
2.11
2.11
1.00

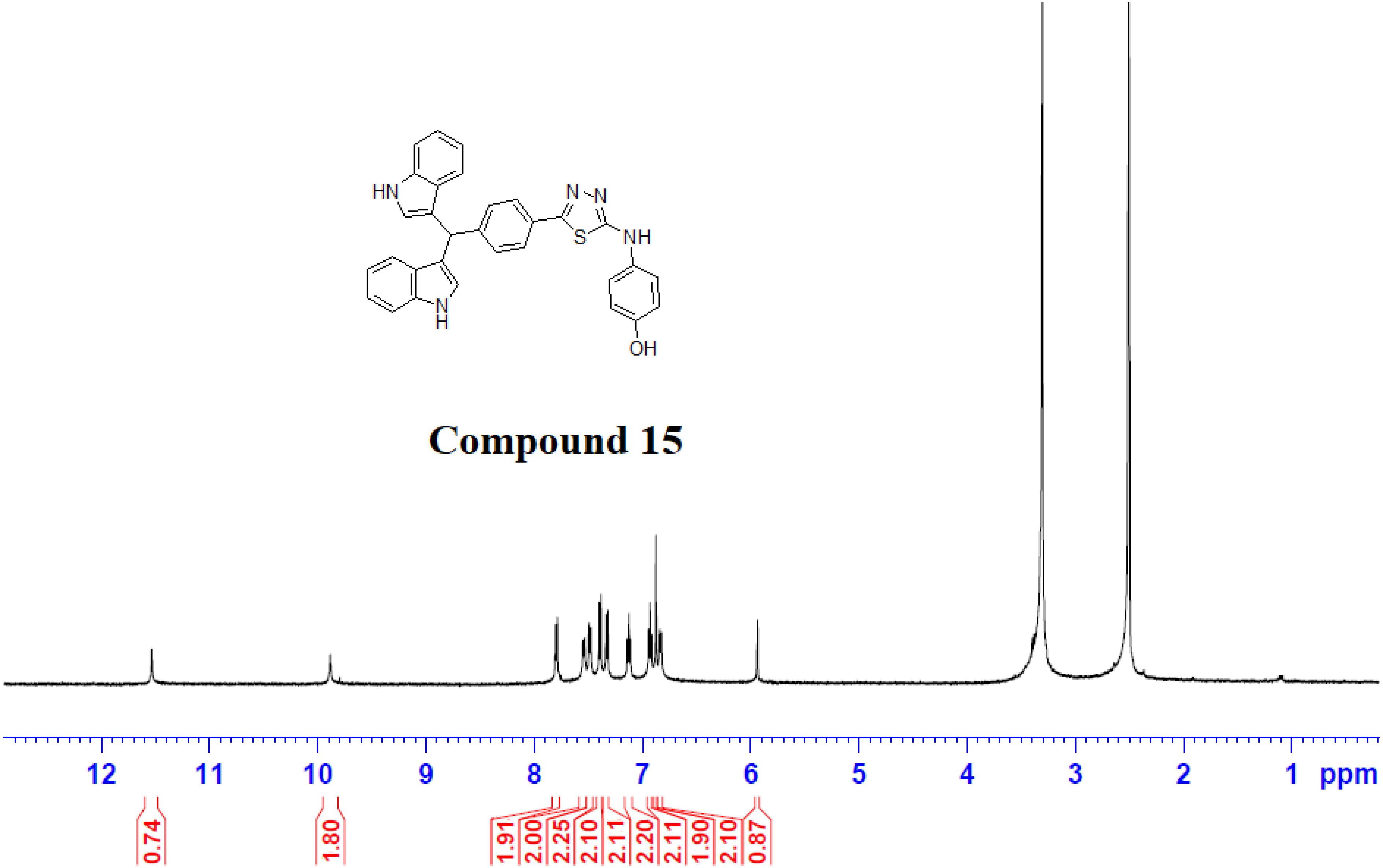
HN
N
N
S
NH
N
H
OH
12 11 10 9 8 7 6 5 4 3 2 1 ppm
0.74
1.80
1.91 2.00 2.25 2.10 2.11 2.20 2.11 1.90 2.10 0.87

Compound 15

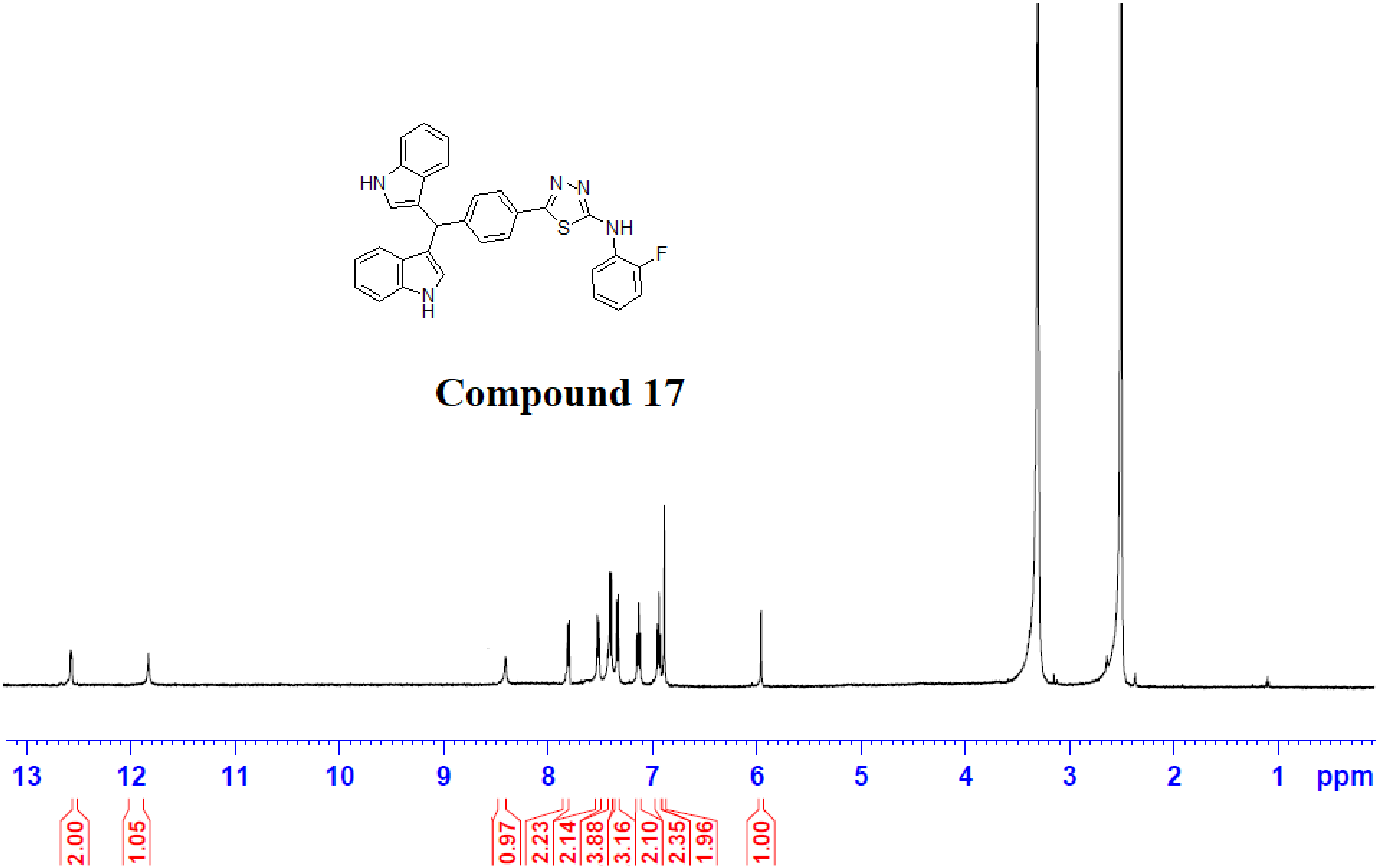

HN
N
N
S
NH
F
N
H
Compound 17
13
12
11
10
9
8
7
6
5
4
3
2
1
ppm
2.00
1.05
0.97
2.23
2.14
3.88
3.16
2.10
2.35
1.96
1.00

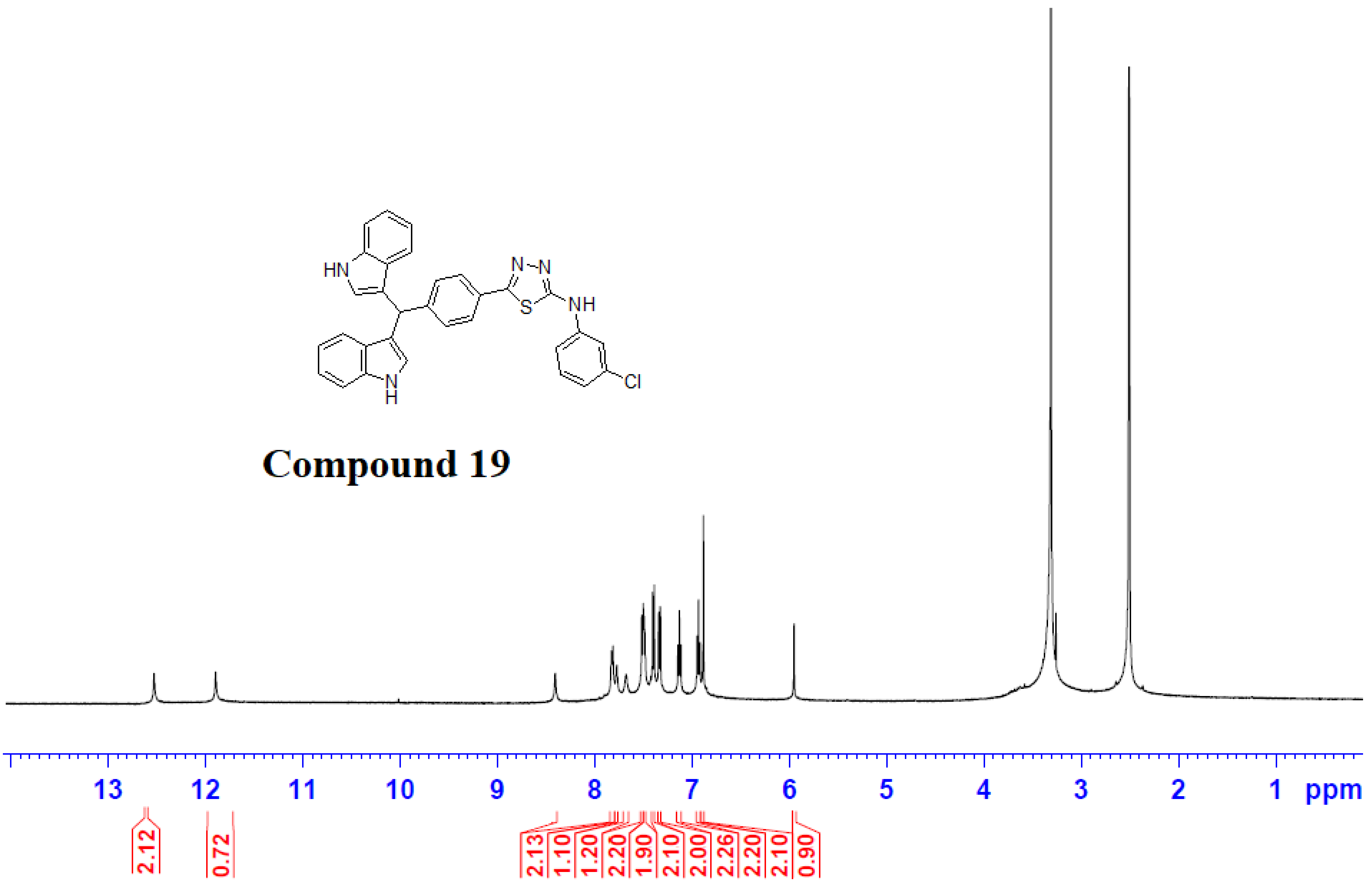
HN
N-N
S
NH
Cl
N
H
Compound 19
13
12
11
10
9
8
7
6
5
4
3
2
1
ppm
2.12
0.72
2.13
1.10
1.20
2.20
1.90
2.10
2.00
2.26
2.20
2.10
0.90

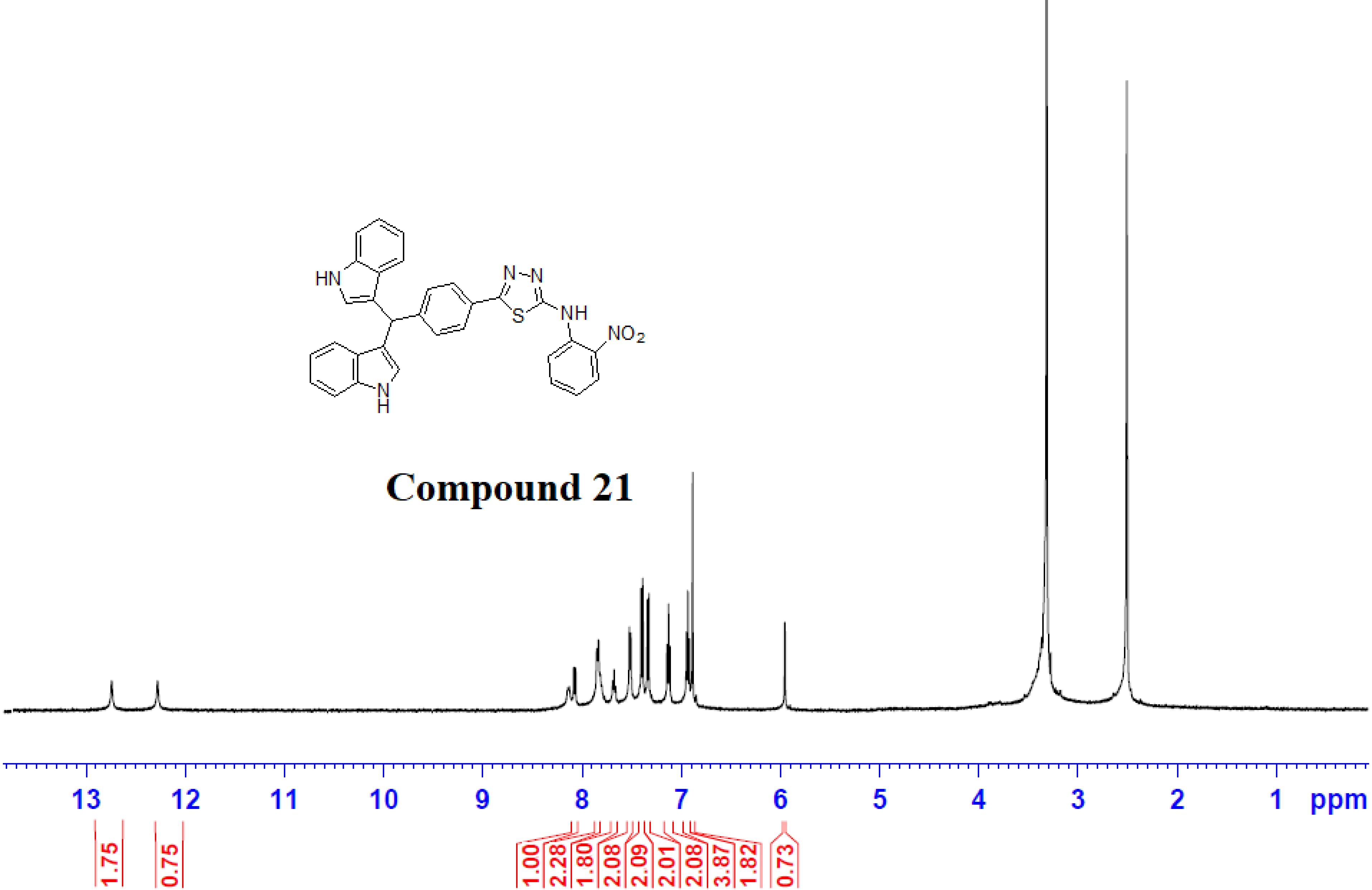

HN
N–N
S
NH
NO2
N
H
Compound 21
13
12
11
10
9
8
7
6
5
4
3
2
1
ppm
1.75
0.75
1.00
2.28
1.80
2.08
2.09
2.01
2.08
3.87
1.82
0.73

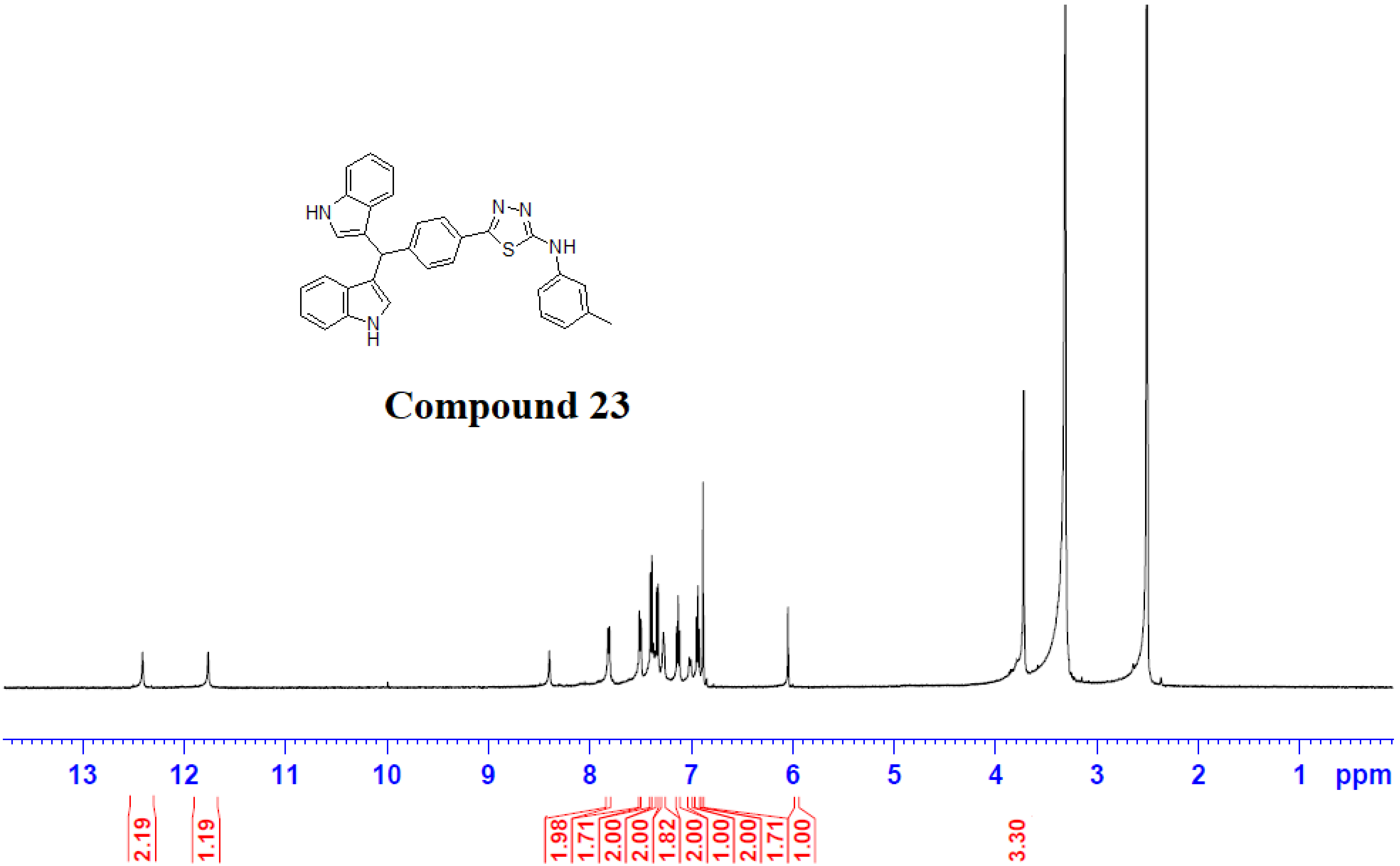
HN
N-N
S
NH
N
H
Compound 23
13
12
11
10
9
8
7
6
5
4
3
2
1
ppm
2.19
1.19
1.98
1.71
2.00
2.00
1.82
2.00
1.00
2.00
1.71
1.00
3.30

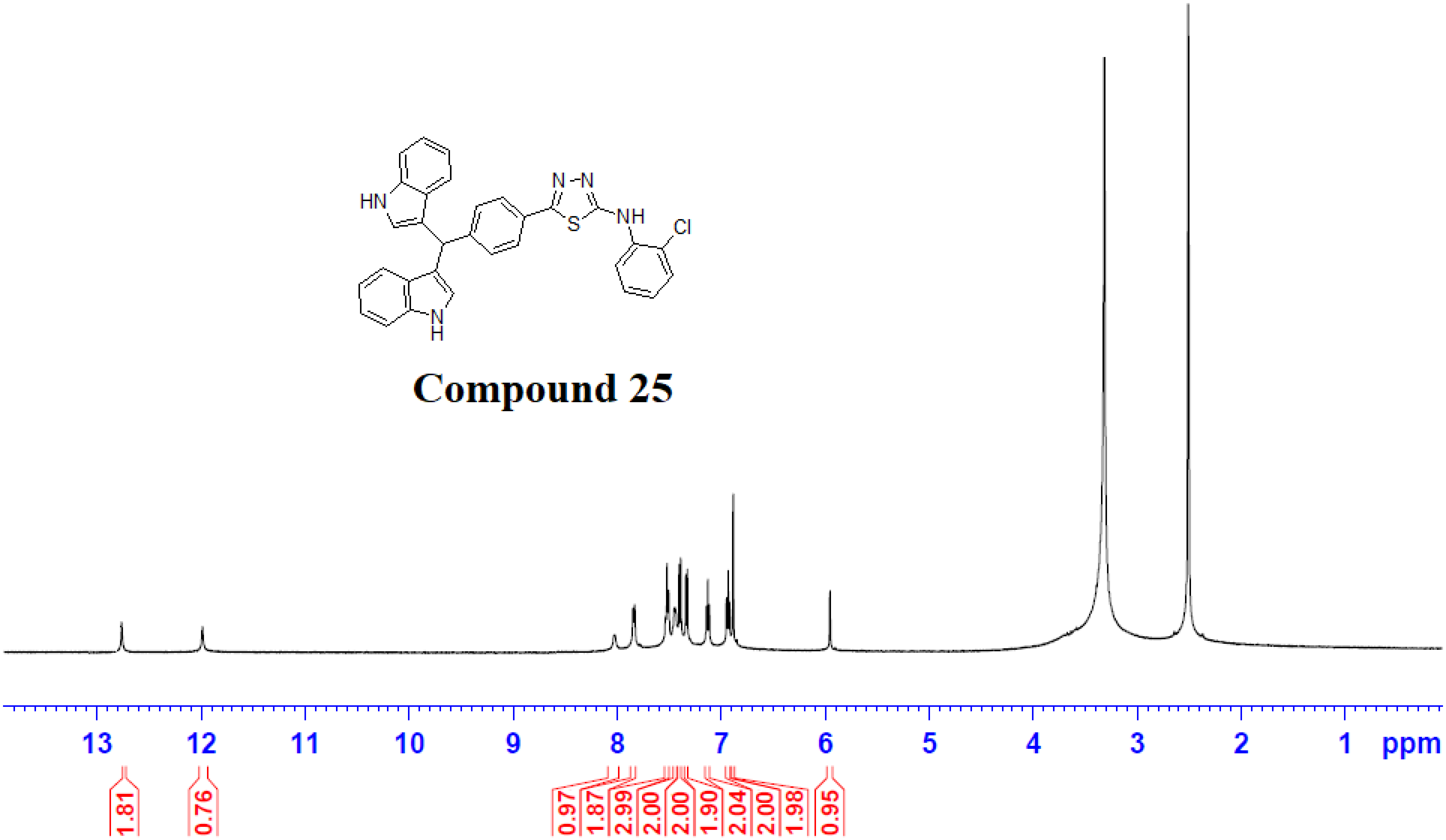

HN
N-N
NH
S
Cl
N
H
Compound 25
13
12
11
10
9
8
7
6
5
4
3
2
1
ppm
1.81
0.76
0.97
1.87
2.99
2.00
2.00
1.90
2.04
2.00
1.98
0.95

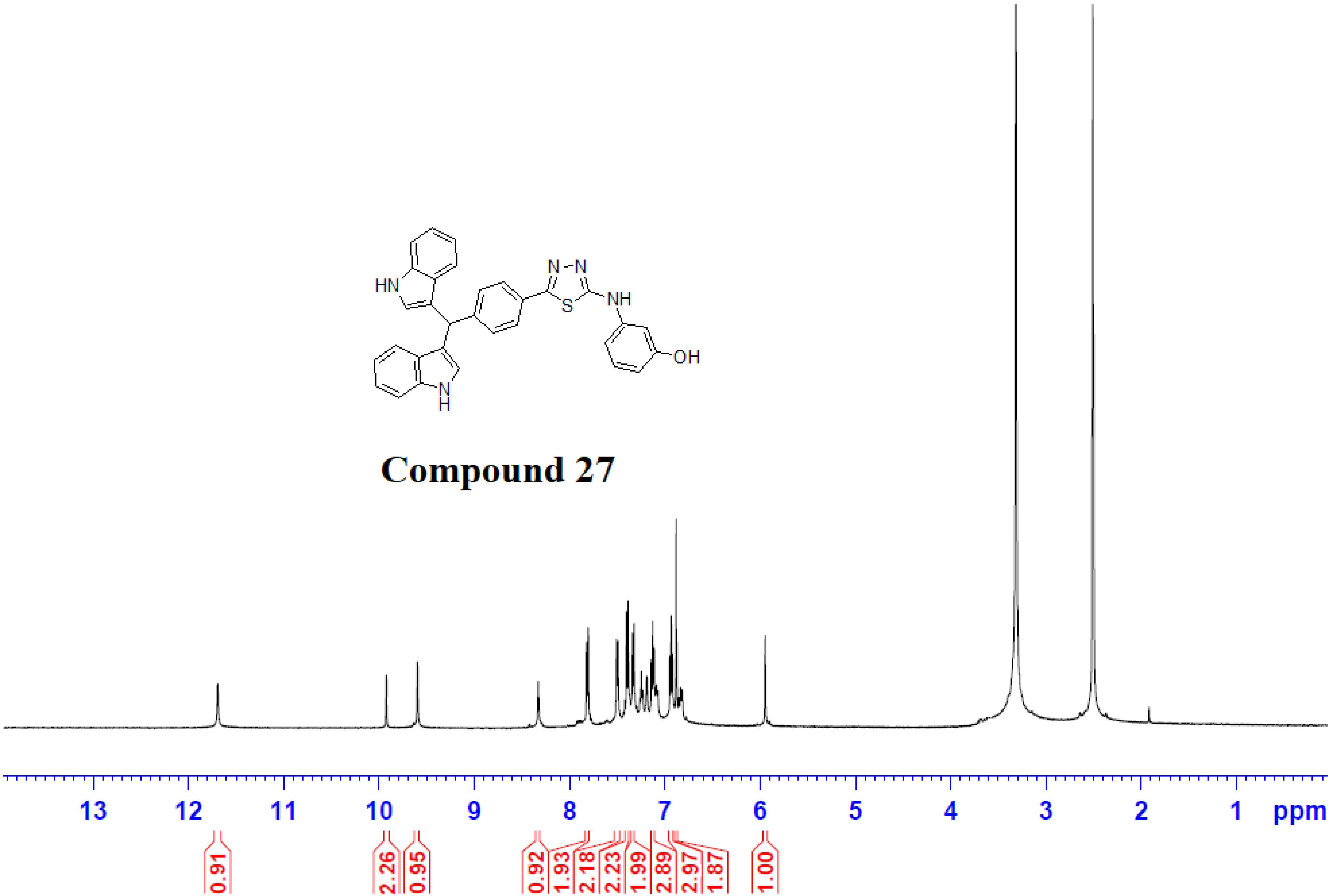
HN
N
H
N
N
S
NH
OH
Compound 27
13
12
11
10
9
8
7
6
5
4
3
2
1
ppm
0.91
2.26
0.95
0.92
1.93
2.18
2.23
1.99
2.89
2.97
1.87
1.00
